# Outcome-associated factors in a molecularly defined cohort of central neurocytoma

**DOI:** 10.1007/s00401-025-02894-3

**Published:** 2025-06-11

**Authors:** Maja Krech, Amos Muench, Daniel Teichmann, Peter Kuzman, Abigail K. Suwala, Franziska M. Ippen, Michael Müther, Katharina J. Weber, Katharina Wenger-Alakmeh, Julia Onken, Peter Vajkoczy, Felix Behling, Sven-Axel May, Georgios Ntoulias, Joachim K. Krauss, Oday Atallah, Majid Esmaeilzadeh, Wolf C. Mueller, Frank L. Heppner, Helena Radbruch, Carsten Dittmayer, Werner Stenzel, Arend Koch, David Capper, David Kaul, Werner Paulus, Karl H. Plate, Joachim P. Steinbach, Markus Czabanka, Rudi Beschorner, Andreas von Deimling, Michael Bockmayr, Julia E. Neumann, Sebastian Brandner, Teresa Krieger, Christian Hartmann, Christian Thomas, Leonille Schweizer

**Affiliations:** 1 Department of Neuropathology, Charité - Universitätsmedizin Berlin, corporate member of, Freie Universität Berlin and Humboldt-Universität Zu Berlin, Berlin, Germany; 2 Institute of Pathology, Charité - Universitätsmedizin Berlin, corporate member of, Freie Universität Berlin and Humboldt-Universität Zu Berlin, Berlin, Germany; 3 Paul-Flechsig-Institute of Neuropathology, University Hospital Leipzig, Leipzig, Germany; 4 Department of Neuropathology, Institute of Pathology, Heidelberg University Hospital, Heidelberg, Germany; 5 Clinical Cooperation Unit Neuropathology, German Cancer Research Center (DKFZ), German Consortium for Translational Cancer Research (DKTK), Heidelberg, Germany; 6 Department of Neurology, Heidelberg University Hospital, Heidelberg, Germany; 7 Department of Neurosurgery, University Hospital Münster, Münster, Germany; 8 Neuroscience Center, Neurological Institute (Edinger Institute), Goethe University, Frankfurt, Heinrich-Hoffmann-Straße 7, 60528 Frankfurt Am Main, Germany; 9 Partner Site Frankfurt, and Geran Cancer Research Center (DKFZ), German Cancer Consortium (DKTK), Heidelberg, Germany; 10 University Cancer Center, Goethe University Frankfurt, Frankfurt Am Main, Germany; 11 Frankfurt Cancer Institute (FCI), Frankfurt Am Main, Germany; 12 Institute of Neuroradiology, Goethe University Frankfurt, Frankfurt Am Main, Germany; 13 Department of Neurosurgery, Charité - Universitätsmedizin Berlin, Berlin, Germany; 14 Department of Neurosurgery, Eberhard Karls University Tübingen, Tübingen, Germany; 15 Department of Neurosurgery, Klinikum Chemnitz, Chemnitz, Germany; 16 Clinic for Neurosurgery Rosenheim, Rosenheim, Germany; 17 Department of Neurosurgery, Hannover Medical School, Hannover, Germany; 18 Department of Neurosurgery, Carl Von Ossietzky University Oldenburg, Oldenburg, Germany; 19 German Center for Neurodegenerative Diseases (DZNE) Within the Helmholtz Association, Berlin, Germany; 20 Cluster of Excellence, NeuroCure, Berlin, Germany; 21 Partner Site Berlin, and German Cancer Research Center (DKFZ), German Cancer Consortium (DKTK), Heidelberg, Germany; 22 Department of Radiation Oncology, Charité - Universitätsmedizin Berlin, Corporate Member of Freie Universität Berlin and Humboldt-Universität Zu Berlin, Berlin, Germany; 23 Department of Radiation Oncology, Health and Medical University Potsdam, Potsdam, Germany; 24 Institute of Neuropathology, University Hospital Münster, Münster, Germany; 25 Dr. Senckenberg Institute for Neurooncology, Department of Neurology, Goethe University Frankfurt, Frankfurt Am Main, Germany; 26 Department of Neurosurgery, Goethe University Frankfurt, Frankfurt Am Main, Germany; 27 Department of Neuropathology, University Hospital Tübingen, Eberhard Karls University Tübingen, Tübingen, Germany; 28 Department of Pediatric Hematology and Oncology, University Medical Center Hamburg-Eppendorf, Hamburg, Germany; 29 Research Institute Children’s Cancer Center Hamburg, Hamburg, Germany; 30 Institute of Neuropathology, University Medical Center Hamburg-Eppendorf, Hamburg, Germany; 31 Department of Neurodegenerative Disease, UCL Queen Square Institute of Neurology and Division of Neuropathology, University College London Hospitals NHS Foundation Trust, London, UK; 32 Department of Neuropathology, Institute of Pathology, Hannover Medical School, Hannover, Germany

**Keywords:** Neurocytoma, DNA methylation profiling, Progression-free survival, FGFR3, Radiotherapy

## Abstract

Central neurocytomas (CN) are intraventricular brain tumors predominantly occurring in young adults. Although prognosis is usually favorable, tumor recurrence is common, particularly following subtotal resection (STR). Currently, the risk of progression is evaluated using atypical features and an elevated Ki67 proliferation index. However, these markers lack consistent definitions, raising the need for objective criteria. Genome-wide DNA methylation profiles were examined in 136 tumors histologically classified as CN. Clinical/histopathological characteristics were assessed in 93/90 cases, and whole-exome sequencing was conducted in 12 cases. Clinical and molecular characteristics were integrated into a survival model to predict progression-free survival (PFS). A diagnosis of CN was epigenetically confirmed in 125 of 136 cases (92%). No DNA methylation subgroups were identified, but global DNA hypomethylation emerged as a hallmark feature of CN associated with higher recurrence risk. Risk stratification based on histological features of atypia and Ki67 proliferation index was not reproducible across neuropathologists. Hypomethylation at the *FGFR3* locus, accompanied by increased FGFR3 protein expression, was observed in 97% of cases. Gross total resection was associated with significantly improved PFS compared to STR, while patients undergoing STR receiving radiotherapy had a better outcome (*p* = 0.0001). Younger patients were identified as having a higher risk of recurrence (*p* = 0.026). Patient age and treatment strategy were key factors associated with survival outcomes in this cohort. These findings underscore the importance of closer follow-up for younger patients and radiotherapy for STR cases. Furthermore, *FGFR3* represents a hallmark feature and potential therapeutic target, warranting further investigation.

**Supplementary Information:**

The online version contains supplementary material available at 10.1007/s00401-025-02894-3.

## Introduction

Central neurocytoma (CN) is a rare intraventricular tumor mainly affecting young adults (mean patient age: 28.5 years) [1]. The clinical course is usually favorable with 10-year overall survival rates of > 80% [17]. Extent of resection (EOR) is the most important prognostic factor in histologically defined CN cohorts, with patients benefiting the most from gross-total resection (GTR) [29]. However, CN may recur even after complete surgical removal, and in some cases malignant behavior with craniospinal dissemination has been reported [10, 25, 35]. CN is currently classified as a CNS WHO grade 2 tumor [1]. According to the most recent 2021 WHO classification of CNS tumors, the diagnosis of CN is established based on histopathology alone. Several studies have shown increased aggressiveness in case of atypical histological features (e.g. brisk mitotic activity, vascular proliferation, necrosis) and/or a Ki67 proliferation index > 2—4% [4, 17, 30, 34]. In 2021, the WHO classification of CNS tumors introduced methylation profiling as a desirable and/or essential diagnostic criterion in combination with histopathological features for diagnosing and grading certain tumors. Molecular reassessment of histology-based cohorts has revealed misclassification rates as high as 12% [5], prompting refinements in prognostic schemes. For example, DNA methylation profiling enhanced diagnostic accuracy in tumors with ependymoma-like morphology and enabled better risk stratification, whereas the histology-based WHO CNS grading lost prognostic significance in epigenetically defined ependymoma classes [38].

The standard of treatment for CN is GTR, if feasible. For patients with subtotal resection (STR), adjuvant radiotherapy (aRT) is often recommended, regardless of histopathological or immunohistochemical findings [17, 29]. Although no specific cutoff value has been established for an elevated Ki67 proliferation index, the 2021 WHO classification of CNS tumors indicates that patients with “higher Ki67 (MIB1) index values” and/or atypical features should be considered for aRT [1]. Chemotherapy, on the other hand, has been investigated in only a limited number of cases, and its efficacy, as well as optimal drug combinations, remains uncertain [3, 19].

In this study, we evaluated the associations of clinical parameters with patient outcome and explored the epigenetic landscape of CN. Our findings also highlight lower patient age and STR (compared to GTR) as significant negative prognostic factors, with patients undergoing STR showing improved outcomes with aRT. Additionally, the consistent upregulation of FGFR3 in our CN cohort suggests a potential avenue for alternative treatment strategies beyond radiotherapy.

## Material and methods

### Patient cohort

Tumor specimens from patients with a histological diagnosis of CN were collected from the archives of the neuropathology departments at the Charité-Universitätsmedizin Berlin, the university hospitals Frankfurt am Main, Hannover, Hamburg, Heidelberg, Leipzig, Münster and Tübingen as well as from the University College London Hospitals NHS Foundation Trust as part of the BRAIN UK, which is supported by Brain Tumor Research and has been established with the support of the British Neuropathological Society and the Medical Research Council [26]. Clinical data, including patient demographics, radiological findings, treatments, and follow-up information, were retrieved from institutional databases or by contacting the treating physicians. EOR was assessed using postoperative MRI and was defined as STR in the presence of residual tumor and GTR if no tumor was detectable. Ethical approval was obtained from the ethics committees of the Charité Berlin (EA1/141/21) and the UCT Frankfurt (UCT-38–2022).

### Immunohistochemical procedures and histopathological review

Formalin-fixed paraffin-embedded (FFPE) tissue from 90 cases was available for analysis. Immunohistochemical stains were performed using primary antibodies against MIB-1 (Ki67, 1:100, clone M7240, Dako, RRID:AB_2142367) and FGFR3 (1:200, clone B9, Santa Cruz Biotechnology, RRID:AB_627596), following standard antigen retrieval protocols. Histological parameters, including tumor necrosis, vascular proliferation, tumor calcification, mitotic count (per mm^2^ in 10 randomly selected high-power fields at 400 × magnification), and the global and focal Ki67 proliferation index, were reviewed by a board-certified neuropathologist (LS).

Eight neuropathologists assessed the total Ki67 index (total Ki67) and the area with the highest Ki67 index (focal Ki67), while five neuropathologists reviewed atypical histological features, including necrosis, vascular proliferation, and mitotic count. Interobserver agreement was evaluated using Krippendorff’s alpha for nominal variables and the intraclass correlation coefficient (ICC3) for continuous variables. To assess interlaboratory variability, seven CN cases were stained at six different neuropathological institutions, and Ki67-positive nuclei were manually counted within a 0.1 mm^2^ area on digitized slides. Details are provided in the Supplementary Methods.

### DNA Methylation and CNV analysis

DNA was isolated from FFPE material using the Maxwell® RSC FFPE Plus DNA Kit (Promega). Methylation analysis was performed with the Illumina Infinium Methylation EPIC BeadChip (n = 129 tumor samples), Illumina Infinium Methylation EPICv2 BeadChip (n = 1) or the Illumina Infinium Human Methylation 450 k array (n = 14), as previously described [37]. Data was analyzed using the R minfi package v1.30.0 and Noob preprocessing [11]. Beta-values were used for consensus clustering using cola v2.4.0 [13]. Additional analysis and clustering methods are detailed in the Supplementary Methods.

Copy number variation (CNV) profiles were calculated from raw DNA methylation data (idat files) using molecularneurolopathology.org, which relies on the R conumee package v1.9.0. Tumors were classified using the Heidelberg Brain Tumor Classifier v12.8 (molecularneuropathology.org). Cases were rated as classifiable based on a classification cutoff value of > 0.9 [6].

### Whole-exome sequencing

Whole-exome sequencing was performed on twelve FFPE tumor samples and four matched blood samples using the TWIST Core Exome and Ref-Seq Kit on the Illumina NextSeq platform (paired-end, 2 × 75 bp) as previously described [36]. Sequencing data were analyzed with a customized bioinformatic pipeline as previously described [24].

### Statistical analysis

The initial diagnosis was defined as the date of first tumor appearance on MRI or, if unavailable, the date of first surgery. Overall survival (OS) was defined as the time from initial diagnosis until death or last contact. Tumor recurrence was defined by either the progression of residual tumor following STR or the reappearance of the tumors on imaging after GTR. Progression-free survival (PFS) was determined as the period between initial diagnosis and tumor recurrence.

**Fig. 1:**
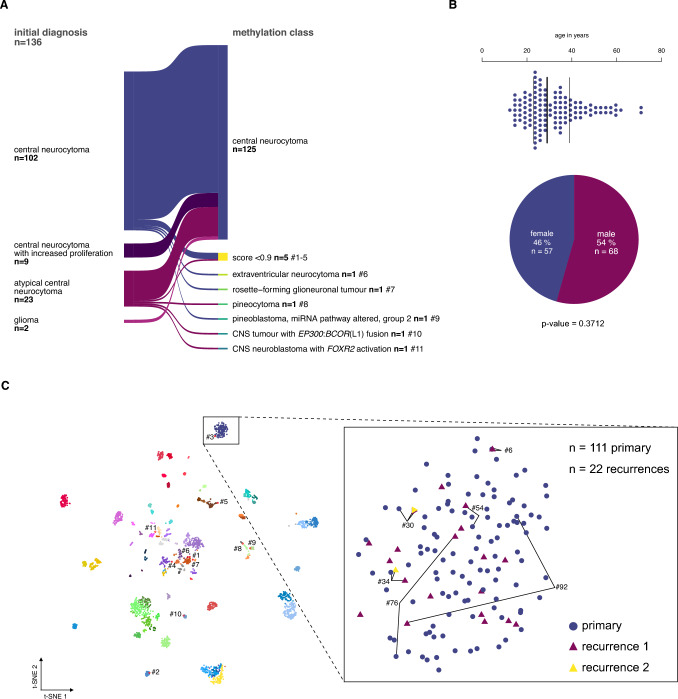
Study cohort selection of the central neurocytoma based on DNA methylation analysis. **A** The DNA methylation profiles of 134 patients with the histopathological diagnosis of CN and two gliomas were analyzed using the Heidelberg Brain Tumor Classifier v12.8 to exclude misdiagnoses. Six cases fell into other methylation classes, and 5 did not yield a sufficient classifier score (< 0.9). **B** The median age at diagnosis of the remaining cases was 29 years (n = 123, NA n = 3). The sex distribution was equal. **C** In the *t*-distributed stochastic neighbor embedding (*t*-SNE) representation, 111 primary and 22 recurrent CNs, including six matched pairs, formed a distinct group separate from other methylation classes of the reference data set from Capper et al. 2018. Cases that have been excluded based on the brain tumor classifier result (red outlines) mapped mostly to other groups, except for one case (#3), which had a prediction score < 0.9 and localized adjacent to the CN group (for consistency excluded from further analyses)

**Fig. 2:**
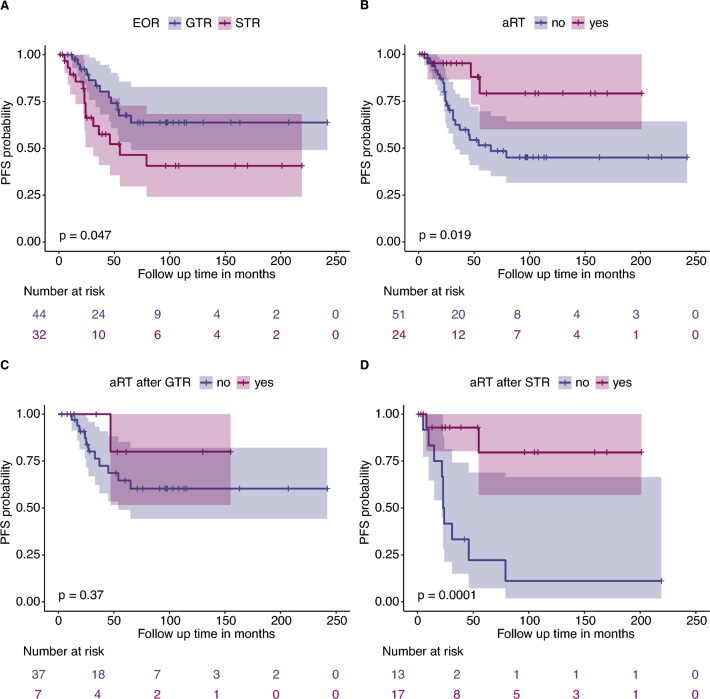
Adjuvant radiotherapy is assocatiated with lower recurrence risk in subtotally resected patients. **A-B** Gross total resection (GTR; *p* = 0.047) and adjuvant radiation therapy (aRT, *p* = 0.019) are significantly associated with better outcome in CN patients. **C-D** However, the advantage of adjuvant radiation was limited to patients with a subtotal resection (STR group, *p* = 0.0001), with no significant effect on PFS for GTR patients (*p* = 0.37)

Survival analyses were conducted using the R packages survminer v0.4.9 and survival v3.7–0. Univariable prescreening was conducted to prune the set of candidate covariates. For univariate analysis, we applied Kaplan–Meier estimation, assessed using log-rank test (threshold *p* < 0.05) and Cox proportional hazard models. None of the variables showed time dependence according to the test for the proportional hazard assumption [12]. For multivariate analysis, we applied Cox proportional hazard models. We tested whether different baseline hazards for STR and GTR increased the likelihood of the model and found no significant increase. Continuous variables were displayed using contour plots [8], while calibration plots, validation statistics, and a patient-centered nomogram were created using rms v6.9. (Figs. 1 and 2)

## Results

### Cohort compilation based on DNA methylation profiles

**Fig. 3:**
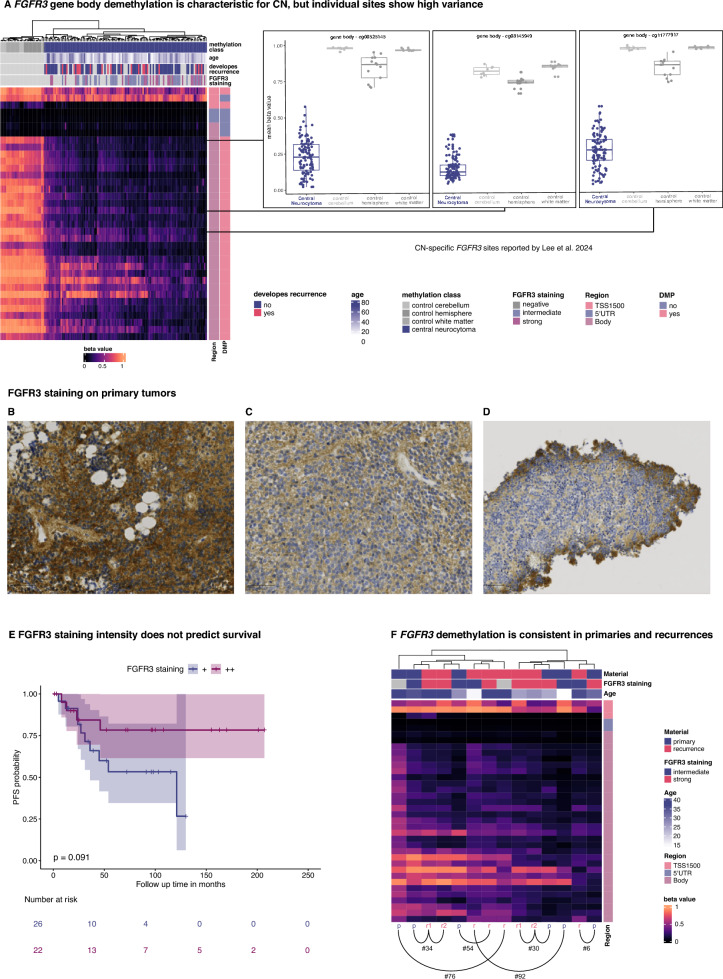
*FGFR3 *gene body hypomethylation and *FGFR3 *overexpression are characteristic for CN.** A** Clustering the methylation values of the *FGFR3* locus of primary tumors and control tissue from Capper et al. 2018 revealed severe hypomethylation at the gene body as a distinct feature of CN. However, individual *FGFR3* CpG sites, especially three sites denoted as CN specific in a previous study by Lee et al. 2024 showed high variance. *FGFR3* methylation levels did not correspond to FGFR3 staining intensity. **B-C** FGFR3 staining intensity ranged from strong in 30/71 (42%) cases to intermediate in 41/71 (58%) cases. **D** FGFR3 immunohistochemistry often demonstrated a gradient effect with stronger staining intensity at the periphery with progressively weaker staining towards the center due to a fixation artifact. **E** Kaplan-Meier estimates did not show a significant PFS difference between cases with strong (+ +) and intermediate (+) staining intensity. **F** *FGFR3* demethylation was consistent across primary and recurrent samples and did not show any association with sample material, the development of a recurrence, or age

The study included 136 tumor samples, of which 134 were histologically diagnosed as CN (including the cohort from Capper et al. [5]) plus 2 samples initially classified as glioma but reclassified as CN after integrated diagnosis (Fig. 1A). Using the v12.8 Heidelberg Brain Tumor Classifier, CN diagnosis was confirmed in 125 of 136 cases (92%). Eleven samples were assigned to another methylation class with a high confidence score (extraventricular neurocytoma, rosette-forming glioneuronal tumor, pineocytoma, pineoblastoma, CNS tumor with *EP300*::*BCOR*(L1) fusion, and CNS neuroblastoma with *FOXR2* activation) or yielded low prediction scores (n = 5; Fig. 3A, Supplementary Table 1).

The epigenetically confirmed CN cohort (125 cases) comprised 111 primary and 22 recurrent samples (of which 8 were matched with primary samples in this series, including 2 samples from the second recurrence). Within this epigenetically validated cohort, the median patient age at initial diagnosis was 29 years (range: 12–72 years, Fig. 1B), and distribution among sexes was equal (female = 57, male = 68: p-value = 0.3712; Fig. 1B).

Upon *t*-distributed stochastic neighbor embedding (*t*-SNE) of all samples with reference data from Capper et al. 2018, primary and recurrent CN cases formed a distinct group (Fig. 1C). Cases excluded from the CN group mapped to other methylation classes, except for one low prediction score case (case #3) localized adjacent to the CN group. Notably, matched primary and recurrent CN samples maintained their methylation class assignment over time and clustered closely in t-SNE space (Fig. 1C, inlet).

### Clinical characteristics of epigenetically validated CNs

The majority of tumors arose in one of the lateral ventricles (69/110, 63%) and involvement of more than one ventricle was observed in 25% (28/110) of cases (Table 1). Hydrocephalus was present in 38% of patients (24/64) at initial MRI, and one patient exhibited leptomeningeal tumor dissemination. Contrast enhancement was reported in 86% (43/50) of cases.

**Table 1:**
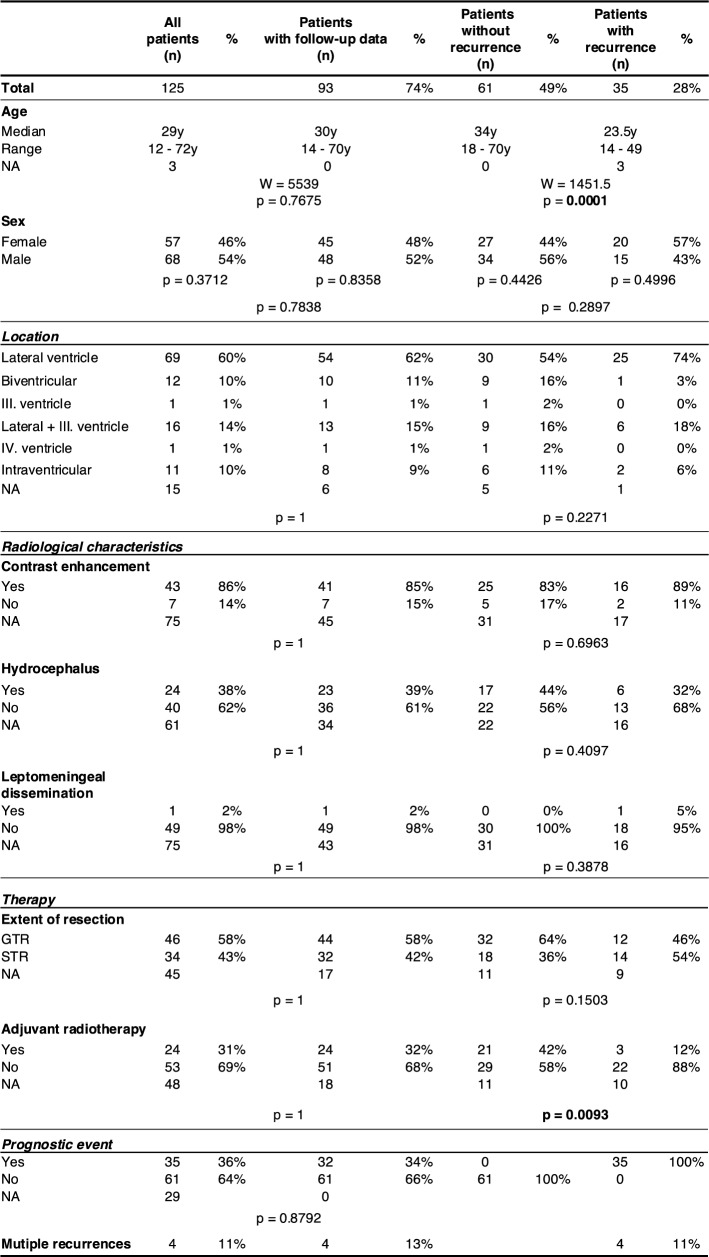
Patient characteristics

GTR was achieved in 46/80 cases (58%), while in 34/80 cases only STR was feasible. aRT was administered in 24/77 patients (31%), predominantly following STR (17/24), though seven patients received aRT after GTR.

Tumor progression was observed in 35/96 patients (37%), with four patients experiencing multiple recurrences. Follow-up data was available for 93/125 patients (74%), and there were no significant differences regarding age, sex, tumor location, radiological characteristics, treatment regimens, and prognostic events between the entire cohort and the subset with available clinical outcome information (Table 1). The median time to tumor recurrence was 30 months (n = 32), with a median follow-up time of 69 months (range 1–357 months, n = 93). One patient died during the follow-up period, though the specific cause of death was not recorded.

Albeit this comparison does not consider observation time, patients experiencing recurrence had a significantly younger age at diagnosis (median age 23.5 years vs 34 years; Mann-Whitney-U-test: W = 1451.5, p-value = 0.0001). These patients were also less likely to have received aRT after initial resection (3/25 vs 21/50, *p* = 0.0093). Patients with recurrence did not differ from patients without recurrence concerning location, radiological characteristics, and EOR (Table 1).

### Differentiation of CNs using atypia criteria lacks prognostic significance

We centrally reviewed the histological features of 89 primary tumors and observed necrosis in 5/89, vascular proliferation in 9/89, and brisk mitotic activity (≥ 1.5 mitoses/mm^2^) in 19/89 cases (average 0.834 mitoses/mm^2^; range 0—4.202 mitoses/mm^2^). One-third of the cases showed intratumoral calcifications (30/89, NA = 35). Necrosis, vascular proliferation, and tumor calcifications individually were also not associated with outcome (Supplementary Table 2 and Supplementary Fig. 1A, C). We stratified the cohort based on the presence of at least one atypical feature cohort into 65 classical CN (cCN) and 24 atypical CN (aCN). However, there was no significant difference in progression-free survival (PFS) between cCN and aCN (Supplementary Fig. 1E, *p* = 0.46, Supplementary Table 2). We also tested whether stratification could be achieved based on the presence of two atypia criteria, which similarly yielded non-significant results (Supplementary Fig. 1F, Supplementary Table 2).

### Centrally reviewed, continuously measured Ki67 index and mitotic count can stratify outcome

Given that the current WHO classification highlights the ongoing debate regarding optimal cutoffs for Ki67 and mitotic counts, we evaluated thresholds of > 2–4% for both global and focal Ki67 within the centrally reviewed subcohort. Global and focal Ki67 values > 2%, as well as focal Ki67 > 3%, were significantly associated with differences in PFS (Supplementary Fig. 2A, Supplementary Table 2).

Similarly, we assessed various cutoffs for mitotic count and identified several thresholds associated with PFS differences in centrally reviewed cases (Supplementary Fig. 4A, B). As the dichotomization of continuous measurements is methodically questionable, we additionally analyzed global/focal Ki67 and mitotic count as continuous variables. All three continuous measures were significantly associated with PFS (Supplementary Figs. 2B, C, 4C). In contrast, neither necrosis nor vascular proliferation demonstrated a significant association with PFS (Supplementary Fig. 1B, D).

### Histological stratification criteria suffer from low reproducibility and prognostic value across multiple raters and laboratories

Followingly, we investigated inter-rater reliability for histological features. Among eight neuropathologists assessing 30 CN cases, agreement was low for necrosis (α = 0.14, range: −0.2 – 0.65, Supplementary Fig. 1B) and vascular proliferation (α = 0.19, range −0.28–1, Supplementary Fig. 1D). Neither necrosis nor vascular proliferation exhibited PFS stratification potential (Supplementary Fig. 1B, D).

Reliability of the Ki67 index assessment by eight neuropathologists was moderate to poor. Total Ki67 showed an ICC3 of 0.55 (Supplementary Fig. 3C), while focal Ki67 had an ICC3 of 0.5 (Supplementary Fig. 3D). Individual raters largely disagreed when estimating the total and focal Ki67 (Krippendorff’s alpha (α) range: −0.3–0.77 and 0.16–0.81); surprisingly, even if raters agreed on a Ki67 hotspot area, their estimations often varied substantially (Supplementary Fig. 3E). Ki67 indices varied across cases, with overall Ki67 ranging from < 1% to 6% (median 2%) and focal Ki67 from < 1% to 12% (median 3%). Across multiple raters, no consistent cutoff for global or focal Ki67 was able to stratify PFS (Supplementary Fig. 3A). Continuous evaluation of global Ki67 stratified PFS in only one rater (rater 1–central reviewer), while focal Ki67 failed to stratify PFS across all raters (Supplementary Fig. 3B).

We assessed inter-laboratory variability in Ki67 staining protocols across six neuropathological centers using serial sections from seven cases. Staining intensity varied markedly between institutions (Supplementary Fig. 3F). To evaluate the diagnostic impact of this variability, we manually counted Ki67-positive cells in a standardized and matched region of 0.1 mm^2^ (Supplementary Fig. 3F, red inset) and applied 2% and 3% cutoffs (as these were significantly associated with PFS in central review) to classify patients into CN with increased proliferation index (iCN). Staining differences led to discordant classification into cCN and iCN in 4/7 cases for both cutoffs (Supplementary Fig. 3G).

Similarly, the range of mitotic counts was high among five raters (Supplementary Fig. 4D) with low inter-rater agreement (α range: −0.03 – 0.65, ICC3 = 0.38, Supplementary Fig. 4E). For mitotic count, two common cutoffs (≥ 1.5/mm^2^ and ≥ 1.9/mm^2^) were associated with PFS differences in only two out of five raters (Supplementary Fig. 4F). Similarly, continuous mitotic count measurements stratified PFS in two raters but not in the remaining three (Supplementary Fig. 4G).

As the histopathological stratification with Ki67, mitotic count, and other atypical features demonstrated poor reproducibility, we did not include them in further survival analysis.

### Adjuvant radiation is associated with improved outcome in subtotally resected patients

Univariate PFS analysis in epigenetically defined CNs confirmed that the EOR is significantly associated with outcome. Patients undergoing GTR had longer PFS than those with initial STR (NA vs 55 months, *p* = 0.049, Fig. 2A). Additionally, patients who received aRT showed improved PFS (Fig. 2B, p = 0.019).

Interestingly, aRT did not significantly prolong PFS in patients with GTR (Fig. 2C, p = 0.37), although only seven patients received aRT following GTR. In contrast, aRT was associated with longer PFS in STR patients (23.5 vs. NA months, no median survival as the survival function does not reach 0.5, *p* = 0.0001, Fig. 2D). Radiological characteristics such as tumor location (uni- vs. multilocular), contrast enhancement (yes vs. no), and the presence of hydrocephalus were not associated with prognosis in Kaplan–Meier or Cox proportional hazard analyses (Supplementary Table 2). The potential effect of primary and residual tumor size on recurrence and the therapy side effects were not evaluated due to absent clinical data.

### No evidence of relevant epigenetic subgroups, chromosomal or genetic alterations associated with outcome in CN

We investigated the potential impact of DNA methylation patterns, chromosomal alterations, and genetic mutations on patient outcomes. Consensus clustering of DNA methylation data from n = 111 primary tumors with top 1 000, 2 000, 5 000 CpG sites revealed a stable partitioning (1-PAC 0.971, SD:mclust k = 2) with 2 subgroups (Supplementary Fig. 5A). However, one of the two groups consisted only of four cases (#19, #65, #96, #98) which did not show distinct clinical or molecular characteristics. Similarly, expanding to the top 10 000, 20 000, and 50 000 CpG sites, we did not find a partitioning with a 1-PAC > 0.9 (Supplementary Fig. 5B). Consensus clustering including recurrent cases only revealed small patient-specific subgroups, suggesting rather unspecific subclusters instead of robust epigenetic subtypes (Supplementary Fig. 5C, D). Thus, CN represent an epigenetically coherent molecular group of tumors, with no evidence of distinct epigenetic subgroups.

Most copy number profiles inferred from methylation data were balanced (119/133 samples), with 11 samples (11/133, 9%) showing individual chromosomal alterations. The most common alteration was gain of chromosome 5 (n = 4/125; 3%; Supplementary Fig. 6D, F, H, I). The 11 samples with chromosomal alterations were comprised of seven primary and four recurrent cases. DNA methylation data for both primary and recurrent tumors was available in six cases. One case developed a partial chromosome 17 gain in the recurrent tumor (Supplementary Fig. 6G), while no copy number changes occurred in the other pairs. We did not see a prognostic association of CNV-altered cases versus CN with balanced CNV profiles (p = 0.51; Supplementary Fig. 6M) or the total CNV load (Cox model p = 0.25, based on 75 cases, Supplementary Table 2) in CN. The MGMT promoter was unmethylated in nearly all primary (110/111; 99%) and recurrent tumors (21/22; 95%).

To identify pathogenic variants in CN, we performed whole-exome sequencing of 12 CN cases. Mean exome coverage was 109X (range 21–235X) and a median of 12 mutations (range 5—22) were detected per case (Supplementary Table 3). Only a fraction of these mutations was classified as “(likely) pathogenic” (median 3, range 1–5) according to ACMG criteria. In 3/12 cases, we found a “likely pathogenic” *BCR* frameshift mutation (present in both splice variants: NM_021574 exon 18: c.3146_3147 insCCGG, p.V1050Rfs*17 and NM_004327 exon 19: c.3278_3279 insCCGG, p.V1094Rfs*17; variant allele frequencies: 17%, 20%, and 35%). Two of the three cases (#6, #13) with a *BCR* mutation developed a recurrence, and case #24 had no evidence of recurrence after 34 months.

### CN demonstrate global hypomethylation, age-dependent methylation and *FGFR3* demethylation

CN primary tumors exhibit significantly lower mean DNA methylation compared to pooled control samples as previously reported [9] (n = 30: white matter n = 9, cerebellum n = 8, hemisphere n = 13; t-test: t = 19.349, p-value ≤ 0.001), and hypomethylation affected methylated regions across the genome (Supplementary Fig. 7A). Overall, we found a drastic global methylation change with 108 790 differentially methylated sites (32% of all sites) in 16 251 genes (79% of all genes, Benjamini–Hochberg adj. p-value < 0.001). Around 18.5% of these sites showed higher methylation levels, while the majority of 81.5% showed decreased methylation (Supplementary Table 4). Among these sites, 35 663 sites (11% gain -, 89% loss of methylation, 45% of all genes) had a noticeable effect size (absolute log_2_ fold change > 0.2). Affected genes were enriched in RHO GTPase cycle, extracellular matrix organization, *NTRK* signaling, and *PI3K*/*AKT* pathways (over-representation analysis, supplementary Fig. 7B). Furthermore, we tested whether age influenced methylation, revealing 7 256 sites in 2 932 genes (adj. p-value < 0.1). To filter tumor-unrelated, age-dependent sites, these results were intersected with cortex data from Kozlenkov et al. [21] revealing that 4% sites and 35% genes were not specific for CN. The remaining 2 631 sites in 1 905 genes showed enrichment for neuronal system, extracellular matrix organization, and *RAF*/*MAPK* signaling (Supplementary Fig. 7C, Supplementary Table 5). Global methylation was not correlated with age (Supplementary Fig. 7D).

As recently described in a small pilot series based on methyl-seq data, *FGFR3* was found to be among the most differentially methylated genes with 76% of the CpGs being hypomethylated in CN [22]. Compared to normal tissue, we noticed a significant DNA demethylation in 31/36 sites of *FGFR3*, mostly located in the gene body (Fig. 3A). However, individual sites described as CN-characteristic by Lee et al. [22] displayed high variance within our cohort. Hypomethylation of *FGFR3* remained constant between primary and recurrent tumors (Fig. 3F). Immunohistochemical staining against FGFR3 was performed in 73 cases and, importantly, overexpression of FGFR3 could be verified in 97% (71/73) of cases compared to a negative staining in normal CNS tissue (n = 5) and in glioblastoma, IDH-wildtype, without *FGFR3::TACC3* fusion (n = 24; data not shown). The staining intensity was moderate in 41/71 cases (58%; Fig. 3B) and strong in 30/71 cases (42%; Fig. 3C). We encountered common staining artifacts such as a lack of FGFR3 staining in thermally altered tissue areas as well as a gradual increase in staining intensity towards tissue edges, likely due to inadequate fixation (Supplementary Fig. 3D). We did not observe significant survival differences in cases with moderate or strong staining intensity (Fig. 3E).

### Predicting recurrence in CN: risk group definition

**Fig. 4:**
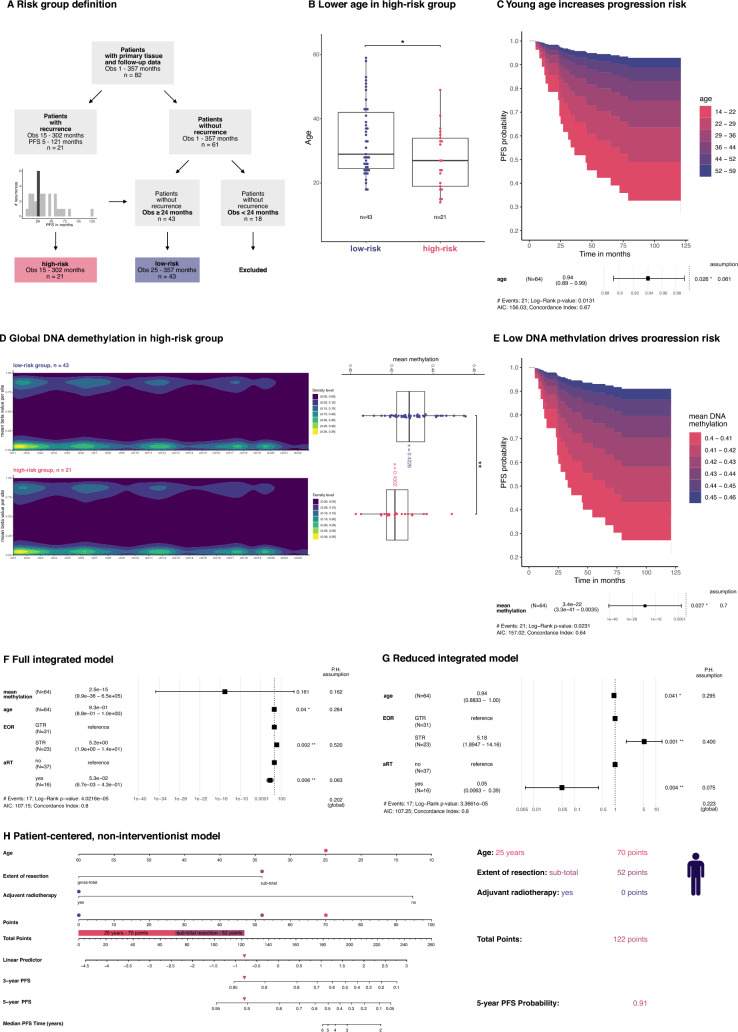
Lower DNA methylation and younger age predict recurrence. **A** Definition of risk groups based on recurrence and observation time. **B-C** The high-risk group was characterized by a significantly younger mean age and young age increases progression risk (P.H. – proportional hazard). Dashed lines indicate PFS probability bounds at the study’s endpoint. **D-E** Mean methylation was significantly decreased across the genome in the high-risk group, and low DNA methylation is a predictor for progression. **F** From a non-interventionist perspective, we first model PFS using mean methylation, age, EOR, and aRT. **G** Followingly, we remove mean methylation as it is not a significant predictor and observe no drastic change in the estimation of coefficients. **H** Visualization of the reduced model in a nomogram with an example calculation

To identify factors that are associated with tumor recurrence, patients with primary tumors and available follow-up data were stratified into two risk groups. Patients who had experienced tumor recurrence were categorized as the high-risk group (Fig. 4A). Notably, most recurrences occurred around 24 months post-diagnosis. To define the low-risk group, patients with an observation period of less than 24 months were excluded to minimize bias from short follow-up durations. The resulting cohort of 64 cases had balanced observation times in the high-risk group (n = 21 with recurrence) and the low-risk group (n = 43 without recurrence; Supplementary Fig. 8A, Supplementary Table 6).

### High-risk CNs are characterized by decreased global methylation and lower age at diagnosis

Joint consensus partitioning of DNA methylation from both risk groups did yield two stable subgroups, albeit with only low sample numbers per class that did not reflect any risk group (Supplementary Table 7). While low-risk and high-risk tumors did not differ at any specific DNA methylation site (including the *FGFR3* locus, Supplementary Fig. 8C), comparing mean methylation in both groups yielded a significantly reduced global methylation in the high-risk group (global beta value 0.4226 vs. 0.4302, t-test: p-value = 0.0097, Fig. 4D). Attempts to classify the groups using a Support Vector Machine failed to achieve separation (balanced accuracy: 0.5).

Age differences between risk groups were notable, with the low-risk group displaying a significantly higher mean age (29 vs. 27 years, W = 396, *p* = 0.0018**,** Fig. 4B, Supplementary Table 6). However, no correlation was observed between age and mean methylation levels (Supplementary Fig. 8B). Only one CpG site with age-dependent methylation was identified within the risk groups, and testing for an interactive effect of age and risk group yielded just two significant sites (Supplementary Table 8).

To further explore the relationship between (chronological) age and DNA methylation, we applied three epigenetic clocks [14, 15, 33] that estimate the DNA methylation-based age (DNAm age) using a linear combination of CpG sites. All clocks showed moderate accuracy in predicting chronological age (Supplementary Fig. 9A, D, G). Only the modified “Skin and Blood” Horvath Clock detected the age difference between risk groups (Supplementary Fig. 9B, E, H). Testing for DNAm age acceleration (residuals of predicted age vs. chronological age) revealed no significant differences between risk groups for any of the clocks (Supplementary Fig. 9C, F, I).

### Integrated model to predict progression

Given the observed differences in mean DNA methylation and age between risk groups, we employed univariate Cox regression analysis across the entire cohort of high- and low-risk cases (n = 64; Fig. 4A), identifying both factors as significant independent predictors of PFS (Fig. 4C, E). We then investigated whether age or mean DNA methylation could refine PFS predictions when the EOR was accounted for. In additive models with EOR, both age and mean DNA methylation individually showed borderline non-significant effects (Supplementary Fig. 8E, J). No interaction effect was observed between age and EOR or between DNA methylation and EOR (Supplementary Fig. 8F, K).

aRT was significantly more common in STR patients (11/23, aRT) than in GTR patients (5/31 aRT; *p* = 0.0167). Using GTR without aRT as the reference, we found age significantly predicted PFS across all treatment regimens. STR without aRT significantly increased the hazard compared to GTR without aRT (Supplementary Fig. 8M). However, no progression events occurred in the 5 cases that received aRT after GTR, precluding hazard ratio estimation. STR followed by aRT did not significantly differ from GTR without aRT (p-value = 0.231, HR = 0.28, SE 0.034–2.3). Mean DNA methylation values failed to show a significant effect when considering the entire treatment regimen (Supplementary Fig. 8H).

To provide patient-specific PFS estimates, we developed a multivariate model incorporating all covariates (full integrative model, Fig. 4F). In this model, DNA methylation was not predictive, while age, EOR, and aRT remained significant predictors. Removing mean methylation, which was not significant, did not alter coefficient estimates substantially (reduced integrative model, Fig. 4G). We found the model well-calibrated at 3 and 5 years and validated the predictors internally using repeated cross-validation (Supplementary Fig. 10). The reduced model was visualized in a nomogram with an example calculation (Fig. 4H). This model is not intended as a treatment recommendation, as our retrospective data cannot exclude confounding variables and only describes associations between PFS and clinical variables. Validation in prospective studies is required.

## Discussion

Previous studies on CN often relied solely on histological diagnosis, which may have led to misclassification due to CN’s heterogeneous appearance and similarity to other CNS tumor entities [1, 34, 39]. The advent of DNA methylation profiling has significantly improved diagnostic accuracy [5], as we found a misclassification rate of 4.48% (4.4% other diagnosis, 3.6% unknown diagnosis, low methylation score) within histologically diagnosed CNs. Based on a series of 125 epigenetically confirmed CNs, we investigated the impact of clinical, histopathological, and molecular characteristics on the outcome of CN patients. We demonstrate that previous histology-based diagnostic approaches, such as Ki67 index and mitotic count, offer stratification potential when centrally reviewed but are limited by poor reproducibility and considerable interlaboratory variability. In contrast, we identified two novel prognostic markers—age and mean global DNA methylation level—that can be assessed more reliably. We developed a multivariate prediction model for patient outcome, integrating clinical and molecular features that may help to define optimal treatment strategies for CN patients.

Neuropathological diagnostics aim to stratify patients with a higher intrinsic capacity of tumor recurrence by transforming signs of biological aggressiveness (e.g. growth rate, active angiogenesis, necrotization, molecular alterations) in a grading scheme that helps clinicians to define which patients may profit from adjuvant treatment. In the case of CN, the Ki67 proliferation index and the presence of atypical features are currently considered important histological markers for progression risk. Several previous studies reported an association of these markers with a more aggressive clinical course, suggesting different Ki67 thresholds (> 2–4%) for guiding treatment decisions (e.g. adjuvant treatment or not) [17, 30, 34]. Most of these studies were assembled based on histologically diagnosed cases, likely introducing bias by mixed cohorts and confounding subsequent analysis.

In our cohort, based on a central review, we found cutoffs for Ki67 index and mitotic count significantly associated with outcome, while the histological classification based on the presence of ≥ one or two atypical features was not associated with outcome. Dichotomizing continuous variables such as Ki67 index by introducing cutoff values is methodologically questionable, as it might introduce a loss of information and spurious correlations [31]. Hence, we demonstrated a significant association of continuous Ki67 index and mitotic count with survival. We conducted an extensive multi-rater review to investigate whether these markers are applicable in routine diagnostics. Our analysis demonstrated moderate to low inter-rater reliability for total and focal Ki67 indices as well as for the mitotic counts per mm^2^. There was no reproducible association between PFS and either cutoff-based or continuous assessment of Ki67 index and mitotic count across raters. Atypical features like necrosis and vascular proliferation also showed low agreement among raters. In addition to human-introduced bias, Ki67 suffers from high laboratory-dependent variance due to inconsistent staining protocols. As central review revealed a stratification potential of Ki67 index, further machine-learning approaches that adjust for inter-laboratory differences or are based on standardized staining might alleviate these shortcomings.

Unlike for several other CNS tumors [40], we did not identify stable subgroups based on DNA methylation signature nor did we observe any association of individual CpG sites with risk groups. However, we found that CNs are characterized by global DNA demethylation compared to normal brain tissue and that the extent of demethylation is associated with recurrence. Given the predominant absence of genetic drivers, our findings hint at a potential epigenetic mechanism that decreases DNA methylation globally and thereby drives recurrence. Previous studies have demonstrated that DNA methylation (DNAm) age acceleration can predict survival outcomes in glioma patients, but our analyses did not reveal similar trends in CN [23]. However, all clocks performed poorly in predicting the chronological age, with only one clock reflecting the existing age differences between groups, indicating that the CpG sites used in these clocks may not be well aligned with CN’s biology.

In line with previous reports [29, 39], univariate analysis confirmed that GTR confers a greater survival benefit compared to STR. However, for functional reasons, GTR is not always feasible (in our cohort only 58% of patients). In cases of initial STR, aRT for improvement of local tumor control and PFS is discussed [4, 17, 29]. Here, we confirm a significant association between aRT and outcome for STR patients. Further, we identified age as a significant predictor of PFS.

Numerous studies illustrated that integrating molecular, histological, and clinical characteristics can substantially improve survival prediction [32, 38]. Pohl et al. [28] reported improved survival prediction accuracy in ependymoma patients by incorporating clinical data (including sex, age, and EOR), methylation profiles, and CNV into their regression model. Hence, we constructed a prediction model combining the clinical markers age, EOR, and aRT with the molecular marker DNA hypomethylation, aiming to enhance survival prediction in CN. Our integrated risk model demonstrated that age as well as no aRT following STR (compared to no aRT following GTR) were key factors influencing progression risk. Patients with STR and aRT had no significant survival differences compared to patients with initial GTR without aRT. Though univariate analysis indicates GTR patients did not benefit from aRT, the sample size for this regime was limited. Given the significant side effects of radiation therapy, such as cognitive decline, radiation necrosis, or radiation-induced malignancy [7, 27] it is crucial to weigh risks and benefits carefully, especially at a young patient age. Age at diagnosis itself turned out to be prognostically independent of therapeutic management. Patients of older age were less likely to develop a tumor recurrence, suggesting closer follow-up for younger CN patients might be advisable for early detection of recurrences. Given the retrospective nature of this study, validation of these findings in a prospective clinical trial would be desirable.

Recent research on the molecular background of CN has shed light on its potential origins and drivers. One study has identified radial glial-like cells as the candidate cell of origin and a deviation of their developmental course in CN [22]. We found extensive age-dependent methylation enriched in *MAPK* and *RAF* cascades and report that increased age is associated with a lower risk of CN recurrence. It is therefore tempting to speculate that age-related differentiation of radial glial cells depletes the pools of potential (recurrent) CN precursor cells.

Lee et al. [22] also reported *FGFR3* hypomethylation and overexpression resulting in the upregulation of the *PIK3-AKT* pathway as a potential tumor driver. Here, we could confirm differential methylation of *PIK3-AKT* and *MAPK* signaling compared to normal tissue in a large-scale cohort. Furthermore, our findings demonstrate that *FGFR3* hypomethylation leading to increased FGFR3 protein expression in tumor cells is a distinctive molecular characteristic of CN. Currently, FDA-approved FGFR inhibitors for urothelial cancer and cholangiocarcinomas are used mainly for tumors with FGFR fusion [20]. Besides pan-FGFR inhibitors with known toxicity issues, selective FGFR3 inhibitors (e.g. TYRA-300, LOXO-435) and tetravalent bispecific antibodies with increased specificity may lead to fewer off-target effects and less toxicity compared to pan-FGFR inhibitors and are being explored in phase 1/2 trials and preclinical models [2, 16, 18, 41]. These advances point toward potential targeted therapies for patients with residual or recurrent CN as an alternative to radiotherapy. Future clinical trials are warranted to evaluate the efficacy of FGFR3 inhibitors in CN treatment, particularly for younger adults, for whom avoiding radiotherapy-related toxicity is a priority.

## Supplementary Information

Below is the link to the electronic supplementary material.

Supplementary file 1 (PDF 10786 KB)

Supplementary file 2 (XLSX 84 KB)

Supplementary file 3 (PDF 276 KB)

Supplementary file 4 (XLSX 45 KB)

Supplementary file 5 (XLSX 41787 KB)

Supplementary file 6 (XLSX 1157 KB)

Supplementary file 7 (PDF 144 KB)

Supplementary file 8 (XLSX 10 KB)

Supplementary file 9 (XLSX 11 KB)

Supplementary file 10 (DOCX 57 KB)

## Data Availability

Data availability Methylation data that support the findings of this study have been deposited in the Gene Expression Omnibus (GEO) repository with the accession number GSE288337.
