# Supplementary material for: Outcome-associated factors in a molecularly defined cohort of central neurocytoma
Source: Acta Neuropathol. 2025 Jun 11;149(1):61. doi: 10.1007/s00401-025-02894-3 (PMC12158839; doi:10.1007/s00401-025-02894-3)
Supplement: Supplementary file 1 — (PDF 10786 KB) [file 401_2025_2894_MOESM1_ESM.pdf]

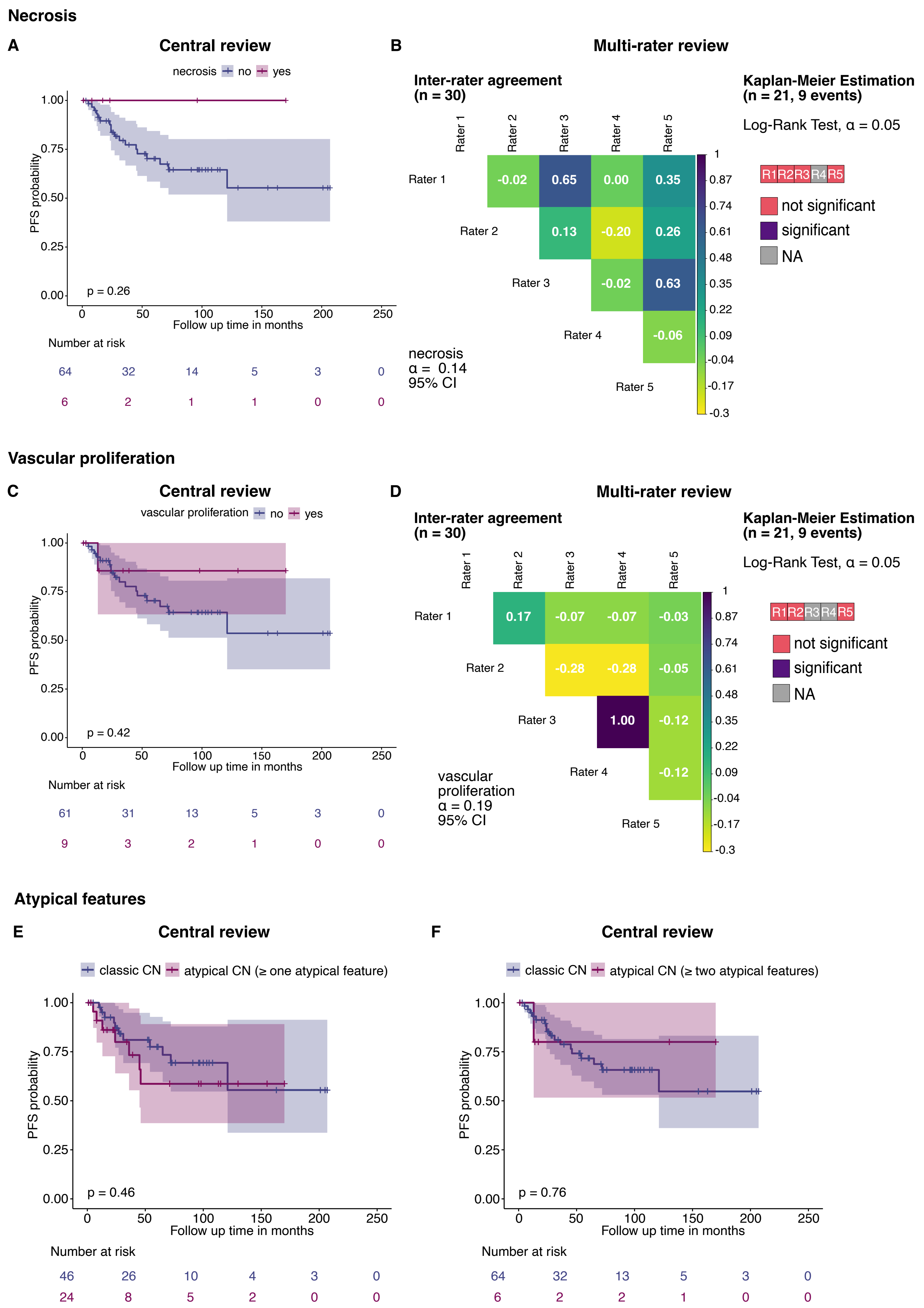
Necrosis
A
Central review
necrosis no yes
PFS probability
1.00
0.75
0.50
0.25
0.00
p = 0.26
0 50 100 150 200 250
Follow up time in months
Number at risk
64 32 14 5 3 0
6 2 1 1 0 0
B
Multi-rater review
Inter-rater agreement
(n = 30)
Rater 1
Rater 2
Rater 3
Rater 4
Rater 5
-0.02 0.65 0.00 0.35
0.13 -0.20 0.26
-0.02 0.63
-0.06
1 0.87 0.74 0.61 0.48 0.35 0.22 0.09 -0.04 -0.17 -0.3
necrosis
α = 0.14
95% CI
Kaplan-Meier Estimation
(n = 21, 9 events)
Log-Rank Test, α = 0.05
R1 R2 R3 R4 R5
not significant
significant
NA
Vascular proliferation
C
Central review
vascular proliferation no yes
PFS probability
1.00
0.75
0.50
0.25
0.00
p = 0.42
0 50 100 150 200 250
Follow up time in months
Number at risk
61 31 13 5 3 0
9 3 2 1 0 0
D
Multi-rater review
Inter-rater agreement
(n = 30)
Rater 1
Rater 2
Rater 3
Rater 4
Rater 5
0.17 -0.07 -0.07 -0.03
-0.28 -0.28 -0.05
1.00 -0.12
-0.12
1 0.87 0.74 0.61 0.48 0.35 0.22 0.09 -0.04 -0.17 -0.3
vascular
proliferation
α = 0.19
95% CI
Kaplan-Meier Estimation
(n = 21, 9 events)
Log-Rank Test, α = 0.05
R1 R2 R3 R4 R5
not significant
significant
NA
Atypical features
E
Central review
classic CN atypical CN (≥ one atypical feature)
PFS probability
1.00
0.75
0.50
0.25
0.00
p = 0.46
0 50 100 150 200 250
Follow up time in months
Number at risk
46 26 10 4 3 0
24 8 5 2 0 0
F
Central review
classic CN atypical CN (≥ two atypical features)
PFS probability
1.00
0.75
0.50
0.25
0.00
p = 0.76
0 50 100 150 200 250
Follow up time in months
Number at risk
64 32 13 5 3 0
6 2 2 1 0 0

Supplementary Figure 1. No prognostic significance and low inter-rater reliability for atypical features in epigenetically defined CN.

A-D) Necrosis ($p = 0.26$) and vascular proliferation ($p = 0.42$) were not associated with PFS in central review and a multi-rater setup. Inter-observer reliability of both parameters was low among five neuropathologists evaluating 30 cases (Krippendorff's alpha values 0.14 and 0.19). **E)** Univariate progression-free survival (PFS) analyses using Kaplan-Meier estimates demonstrate that the presence of at least one atypical feature (e.g. necrosis +/- vascular proliferation +/- brisk mitotic activity of >1.5 mitoses/mm²) based on a central review was not significantly associated with PFS ($p = 0.46$). **F)** Applying a more stringent definition (≥ two atypia criteria) did not stratify PFS either.

Ki67 index - central review

A Kaplan-Meier estimates for PFS with different Ki67 cutoffs

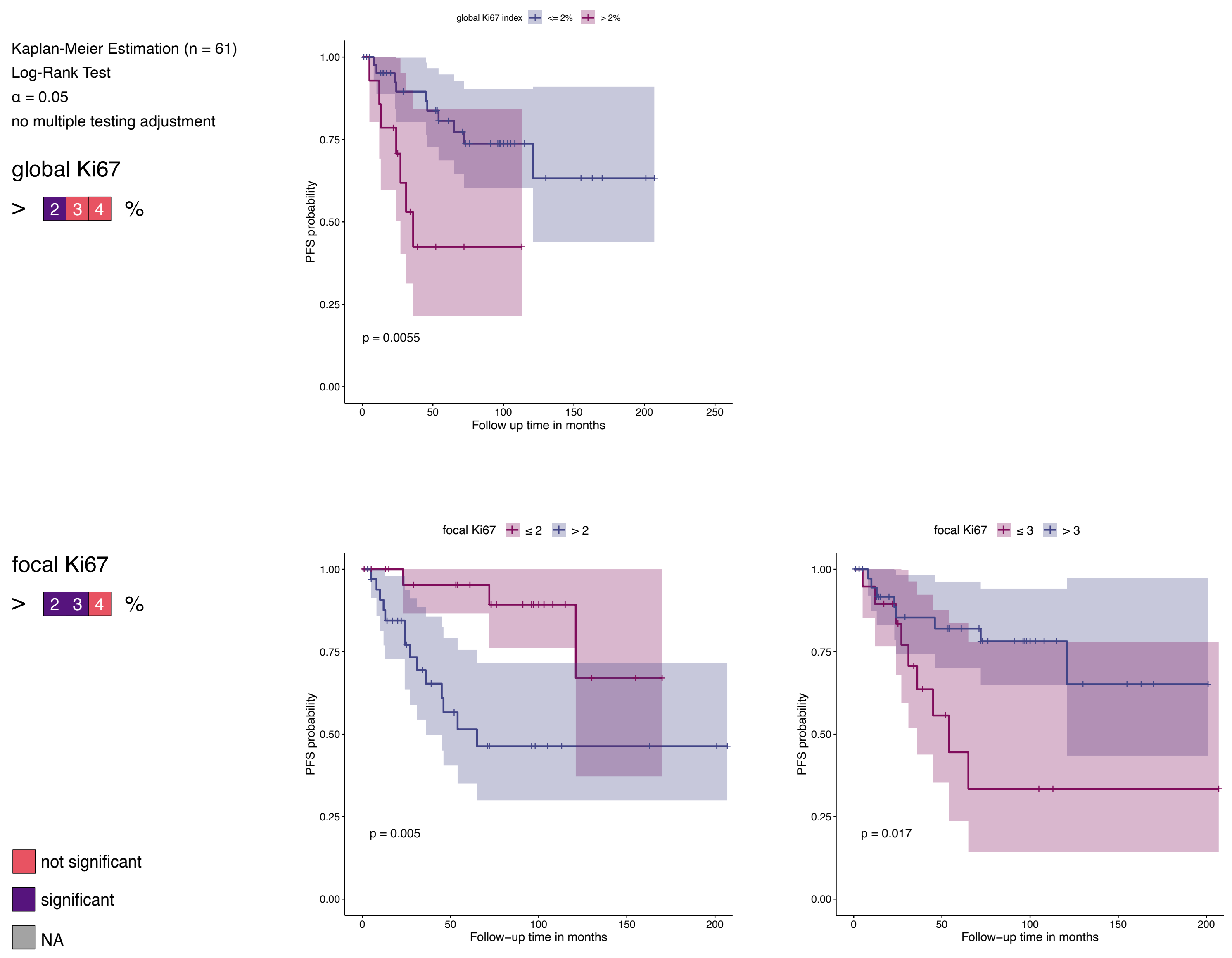

B continuous global Ki67 index

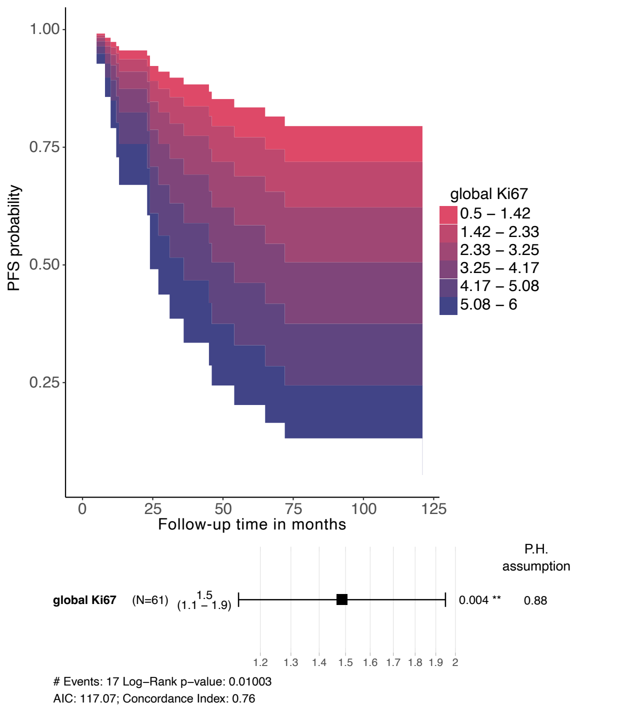

C continuous focal Ki67 index

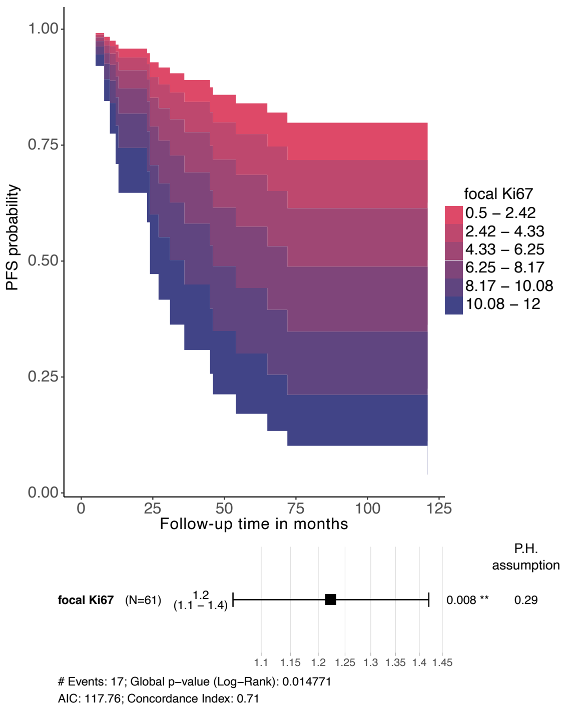

Supplementary Figure 2. Centrally reviewed, continuous Ki67 can stratify PFS.
A) We employed various cutoffs for both global and focal Ki67 index to stratify PFS. Significance was found for cutoffs of 2% for global and focal Ki67, and 3% for focal Ki67. B-C) Continuous global and local Ki67 can stratify outcome in a Cox regression. P.H. – proportional hazard.

Ki67 index - multi-rater review

A Kaplan-Meier estimates for PFS with different Ki67 cutoffs per rater (R1 - R8)

Kaplan-Meier Estimation (n = 21, 9 events)
Log-Rank Test, α = 0.05, no multiple testing adjustment

B Cox regression with continuous Ki67 index per rater (R1 - R8)

Cox proportional hazard model (n = 21, 9 events)
Wald Test, α = 0.05, no multiple testing adjustment

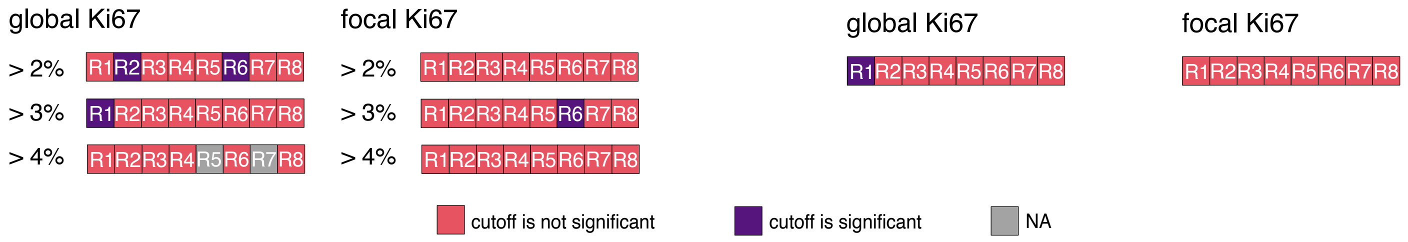

C

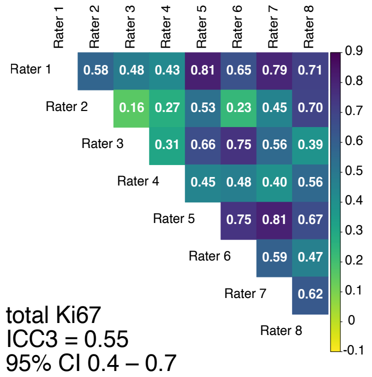

E

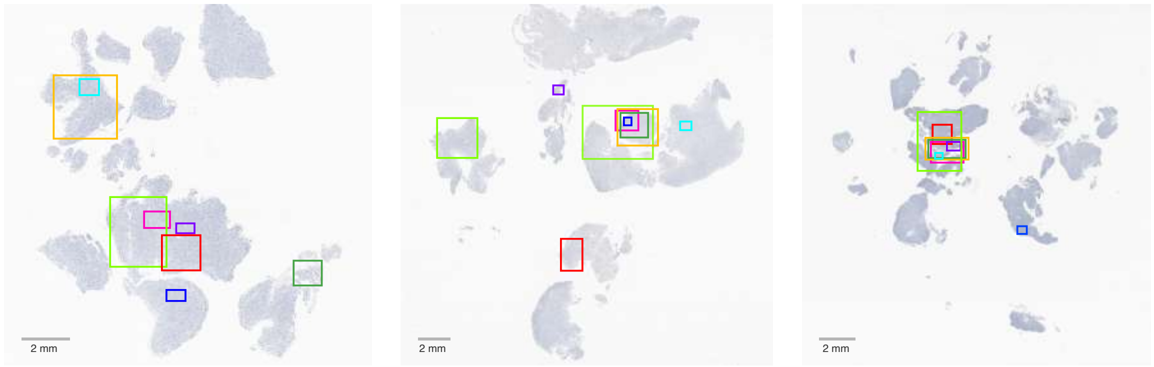

Case 1: focal Ki67 1 - 4% Case 5: focal Ki67 1.5 - 11% Case 9: focal Ki67 1- 10%

D

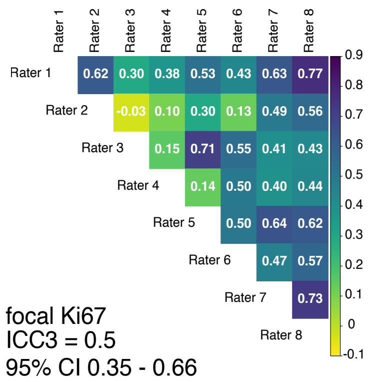

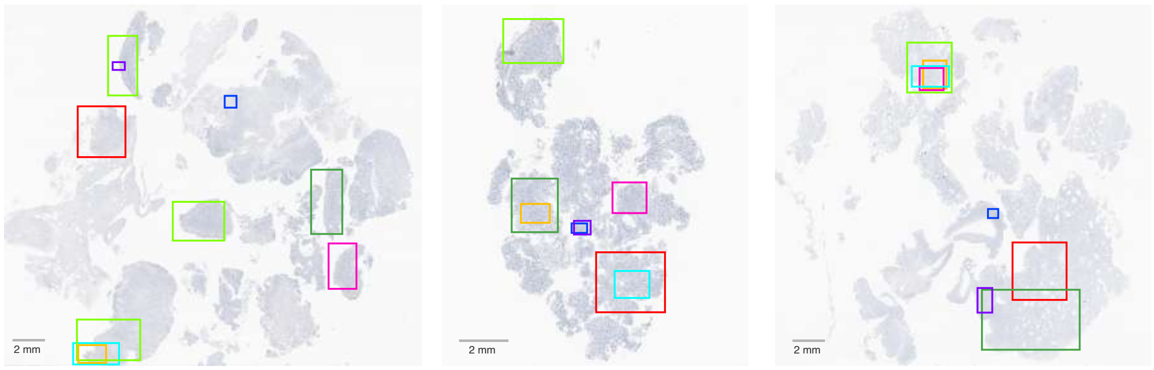

Case 16: focal Ki67 4.5 - 10% Case 19: focal Ki67 1 - 4% Case 30: focal Ki67 2.5 - 8%

Ki67 index - inter-laboratory review

F

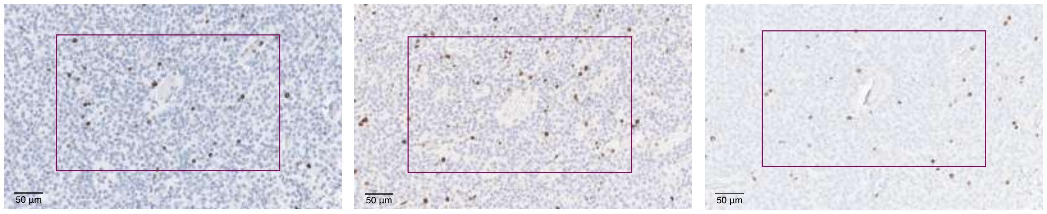

Institution A
Antibody: MIB1 (monoclonal mouse, Dako M7240)
Dilution: 1:100
Stainer: Roche Ventana Benchmark ultra

Institution B
Antibody: MIB1 (monoclonal mouse, Dako M7240)
Dilution: 1:200
Stainer: Leica BOND III

Institution C
Antibody: SP6 (monoclonal rabbit, Cell Marque AB Merk)
Dilution: 1:750
Stainer: Roche Ventana Benchmark XT

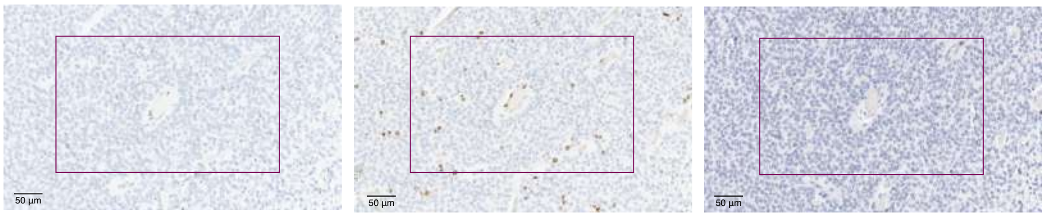

Institution D
Antibody: RTU (monoclonal rabbit, Roche, Clone 30.9)
Stainer: Roche Ventana Benchmark ultra

Institution E
Antibody: MIB1 (monoclonal mouse, Dako M7240)
Dilution: 1:100
Stainer: Roche Ventana Benchmark ultra

Institution F
Antibody: RTU (monoclonal mouse, class II, Dako GA626)
Stainer: Dako Omnis

G

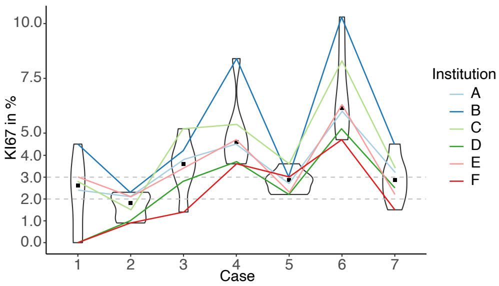

Focal Ki67 cutoff ≤2 % (cCN) / > 2 % (iCN)							
Institution	Case 1	Case 2	Case 3	Case 4	Case 5	Case 6	Case 7
A	iCN	cCN	iCN	iCN	iCN	iCN	iCN
B	iCN	cCN	iCN	iCN	iCN	iCN	iCN
C	iCN	cCN	iCN	iCN	iCN	iCN	iCN
D	cCN	cCN	iCN	iCN	cCN	iCN	cCN
E	iCN	cCN	iCN	iCN	cCN	iCN	cCN
F	cCN	cCN	cCN	iCN	iCN	iCN	cCN

Focal Ki67 cutoff ≤ 3 % (cCN) / > 3% (iCN)							
Institution	Case 1	Case 2	Case 3	Case 4	Case 5	Case 6	Case 7
A	cCN	cCN	iCN	iCN	cCN	iCN	iCN
B	iCN	cCN	iCN	iCN	cCN	iCN	iCN
C	cCN	cCN	iCN	iCN	iCN	iCN	iCN
D	cCN	cCN	cCN	iCN	cCN	iCN	cCN
E	cCN	cCN	iCN	iCN	cCN	iCN	cCN
F	cCN	cCN	cCN	iCN	cCN	iCN	cCN

Supplementary Figure 3. The multi-rater and inter-laboratory evaluation of the Ki67 index demonstrates low prognostic value and reproducibility.

A) No cutoff for global and focal Ki67 could consistently stratify PFS across raters. **B)** Continuous evaluation of global Ki67 only stratified PFS in one rater (rater 1 – central reviewer), while focal Ki67 failed to stratify PFS in all raters. **C-D)** The evaluation of 30 CN cases by eight neuropathologists revealed moderate to poor agreement on the average Ki67 index estimation (total Ki67; ICC3 = 0.55) as well as the region with the highest Ki67 index (focal Ki67; ICC3 = 0.5). **E)** Case examples of the "hotspot" regions chosen by different neuropathologists: even if the agreement over a "hotspot" was high, Ki67 index estimation differed widely between raters evaluating the same slide. **F)** Staining intensity of Ki67 on serial sections was substantially different between six diagnostic centers. RTU - ready to use. **G)** Manual Ki67 counting in a matched region of 0.1 mm^2 (e.g. red inlet in Supplementary Figure 3F) in seven cases revealed a high variability between centers depicted in the violin plots**. H)** Applying a cutoff of > 4%, 4/7 cases would have been inconsistently assigned to the categories classical CN (cCN) and aggressive CN with increased proliferation index (iCN).

Mitotic count - central review

A Kaplan-Meier estimates for PFS with different mitotic count cutoffs

Kaplan-Meier Estimation (n = 70)
Log-Rank Test, α = 0.05, no multiple testing adjustment

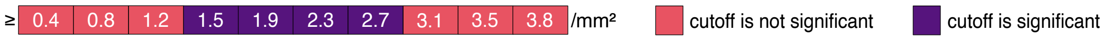

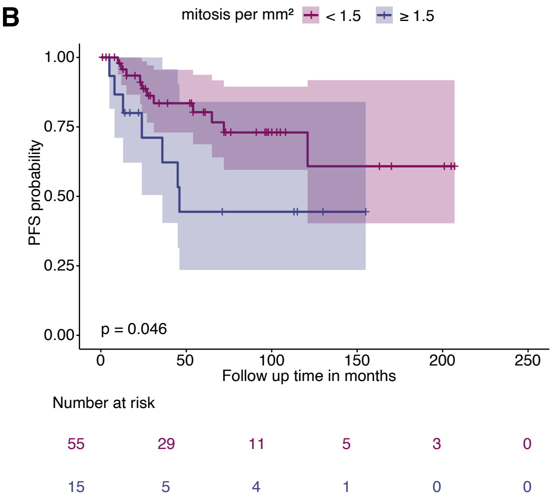

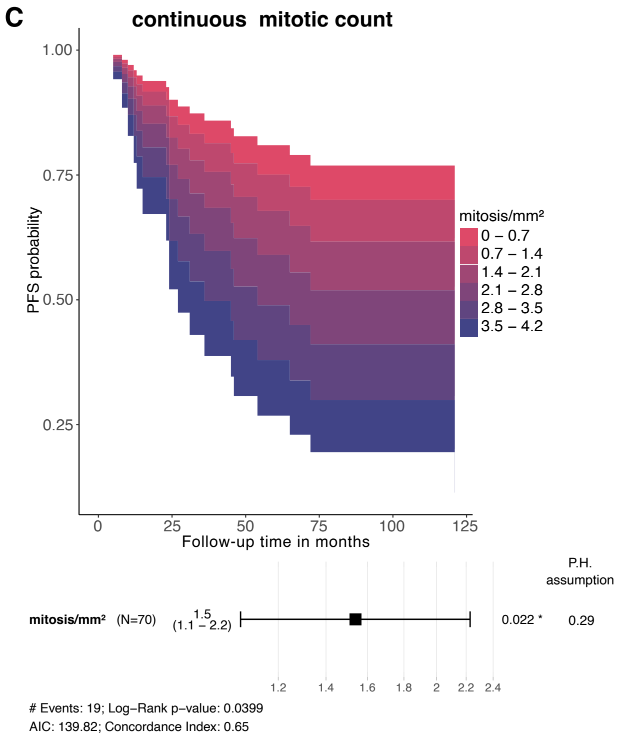

Mitotic count - multi-rater review

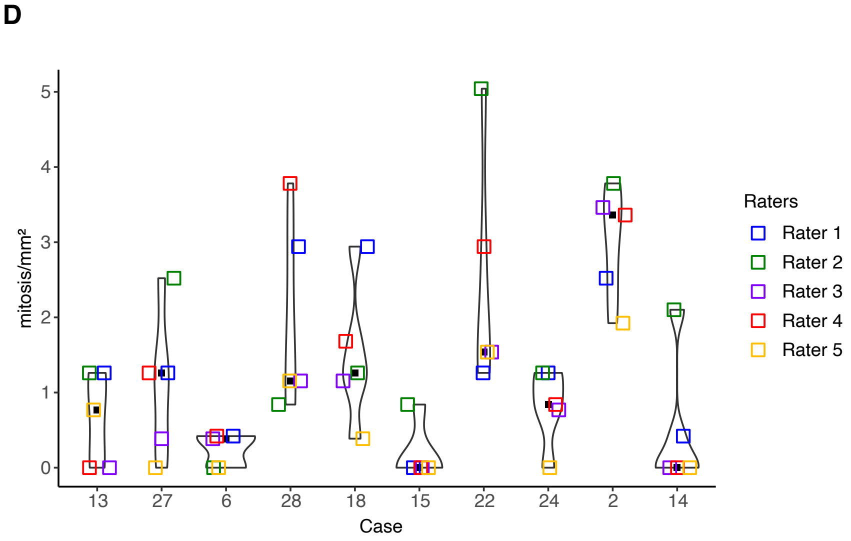

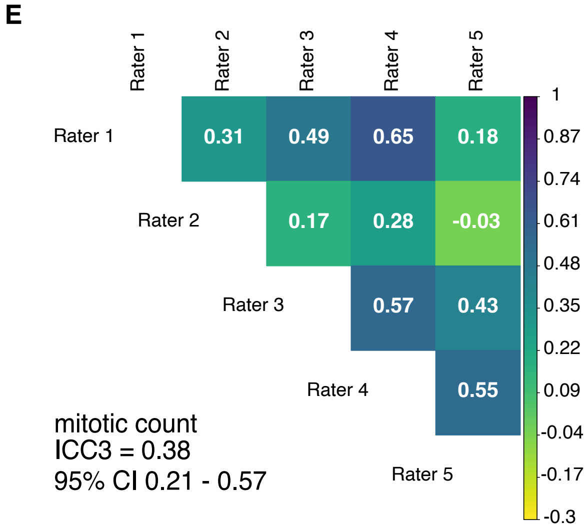

F Kaplan-Meier estimates for PFS with different mitotic count cutoffs per rater (R1 - R5)

Kaplan-Meier Estimation (n = 21, 9 events)
Log-Rank Test, α = 0.05, no multiple testing correction

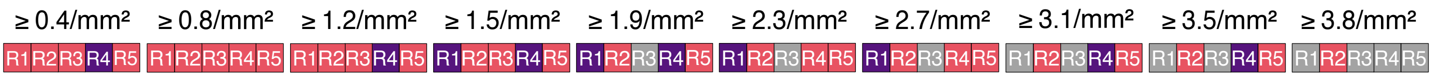

G Cox regression with continuous mitotic count per rater (R1 - R5)

Cox proportional hazard model (n = 21, 9 events)
Wald Test, α = 0.05, no multiple testing correction

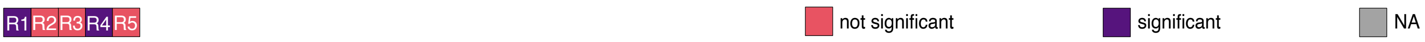

Supplementary Figure 4. Central- and multi-rater review of mitotic count demonstrates low prognostic value and reproducibility.

A-B) Centrally reviewing mitotic count indicated a significant cutoff for ≥ 1.5/mm^2 - ≥ 2.7/mm^2. C) Cox regression using a continuous measurement of centrally evaluated mitotic count can differentiate PFS. D) Violin plots of 10 case examples demonstrated that the range of the mitotic count between raters was high. E) The inter-rater agreement of five neuropathologists evaluating the mitotic count (mitoses/mm2) was also poor (ICC3 = 0.38). **F)** Two common cutoffs for mitotic count (≥ 1.5/mm^2 and ≥ 1.9/mm^2) that could differentiate PFS could be found only in two out of five raters (rater 1 – central reviewer and rater 5). G) Continuous mitotic count can stratify PFS in two raters (rater 1 – central reviewer and rater 4), but not in the other three raters.

A

Primary tumors (n = 111) consensus partitioning, top 1 000, 2 000, 5 000 sites

Top value method: partition method	best k	1-PAC	Mean silhouette	Concordance
<u>SD:mclust</u>	2	<u>0.971</u>	0.948	0.971
CV:mclust	3	0.442	0.689	0.867
CV:hclust_ward_D2	3	0.422	0.681	0.872
MAD:mclust	3	0.394	0.802	0.878
SD:pam	3	0.306	0.703	0.862
SD:skmeans	6	0.306	0.167	0.444
CV:pam	5	0.276	0.539	0.774
MAD:pam	3	0.261	0.664	0.843
SD:hclust_ward_D2	4	0.211	0.522	0.737
CV:skmeans	2	0.111	0.612	0.792
MAD:hclust_ward_D2	3	0.079	0.554	0.775
MAD:skmeans	2	0.059	0.566	0.772
SD:hclust	4	0.043	0.447	0.645
MAD:hclust	3	0.039	0.586	0.775
CV:hclust	NA	NA	NA	NA

Consensus heatmaps for k = 2,...,6

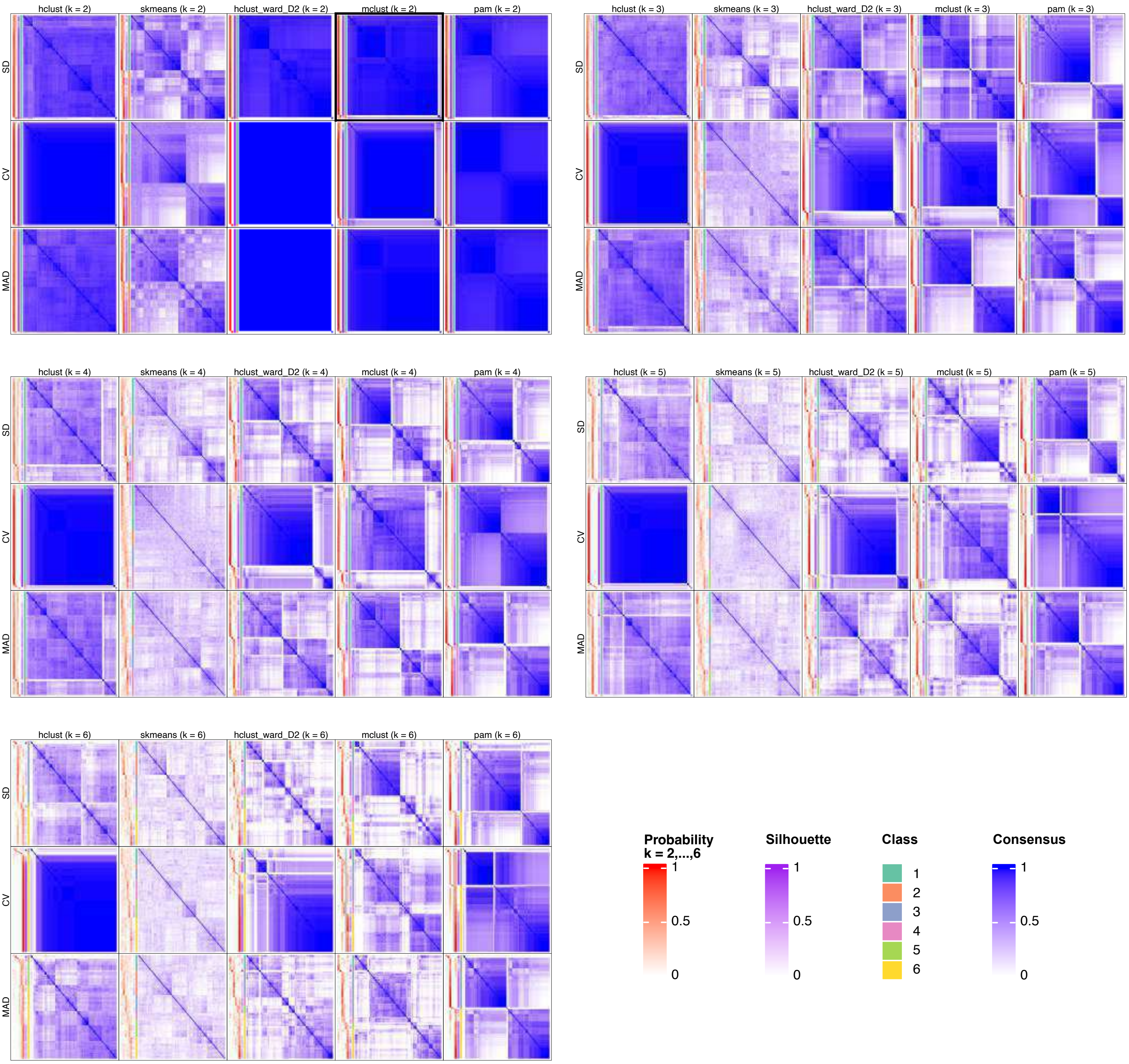

B

Primary tumors (n = 111) consensus partitioning, top 10 000, 20 000, 50 000 sites

Top value method: partition method	best k	1-PAC	Mean silhouette	Concordance
CV:mclust	3	0.789	0.819	0.925
CV:hclust_ward_D2	4	0.545	0.693	0.864
CV:hclust	4	0.496	0.768	0.889
SD:skmeans	2	0.407	0.746	0.878
MAD:skmeans	6	0.352	0.191	0.439
MAD:mclust	4	0.339	0.594	0.757
SD:hclust_ward_D2	3	0.293	0.71	0.85
SD:pam	4	0.29	0.605	0.829
MAD:hclust_ward_D2	3	0.277	0.59	0.782
CV:skmeans	2	0.263	0.684	0.853
CV:pam	5	0.263	0.6	0.745
MAD:pam	6	0.25	0.448	0.779
SD:mclust	3	0.091	0.376	0.724
SD:hclust	4	0.062	0.472	0.731
MAD:hclust	3	0.057	0.57	0.699

Consensus heatmaps for k = 2,...,6

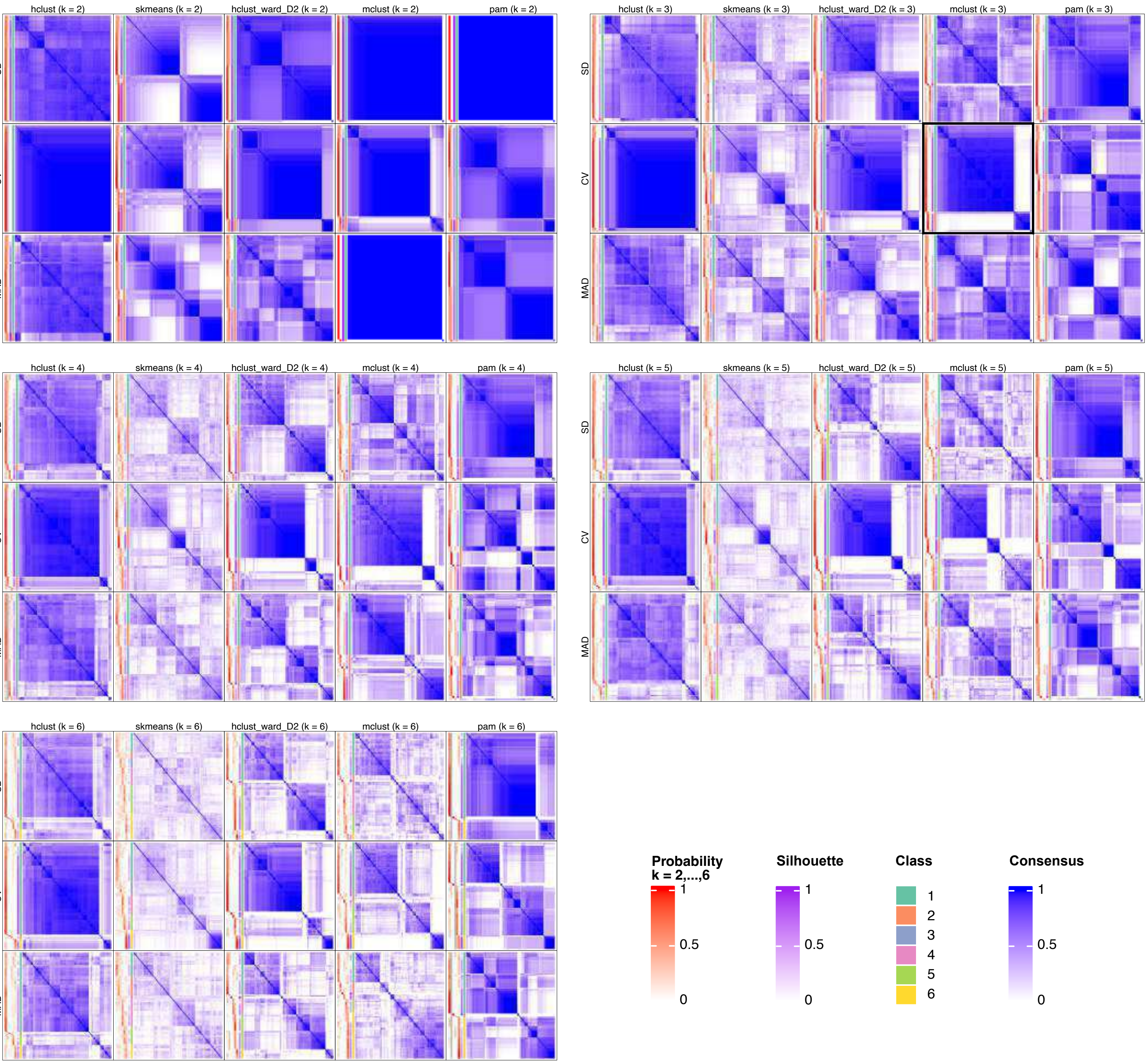

C

Primary (n = 111) and recurrent tumors (n = 22) consensus partitioning, top 1 000, 2 000, 5 000 sites

Top value method: partition method	best k	1-PAC	Mean silhouette	Concordance
SD:mclust	2	1	0.973	0.989
CV:mclust	2	1	0.992	0.995
MAD:mclust	2	1	0.978	0.991
CV:pam	6	0.438	0.617	0.854
CV:hclust_ward_D2	6	0.405	0.668	0.853
MAD:pam	6	0.333	0.652	0.837
SD:skmeans	3	0.253	0.541	0.748
SD:pam	6	0.233	0.626	0.785
SD:hclust_ward_D2	6	0.196	0.438	0.715
CV:skmeans	2	0.146	0.63	0.805
MAD:skmeans	2	0.106	0.614	0.805
MAD:hclust	4	0.044	0.366	0.643
SD:hclust	4	0.042	0.302	0.563
CV:hclust	NA	NA	NA	NA
MAD:hclust_ward_D2	NA	NA	NA	NA

Consensus heatmaps for k = 2,...,6

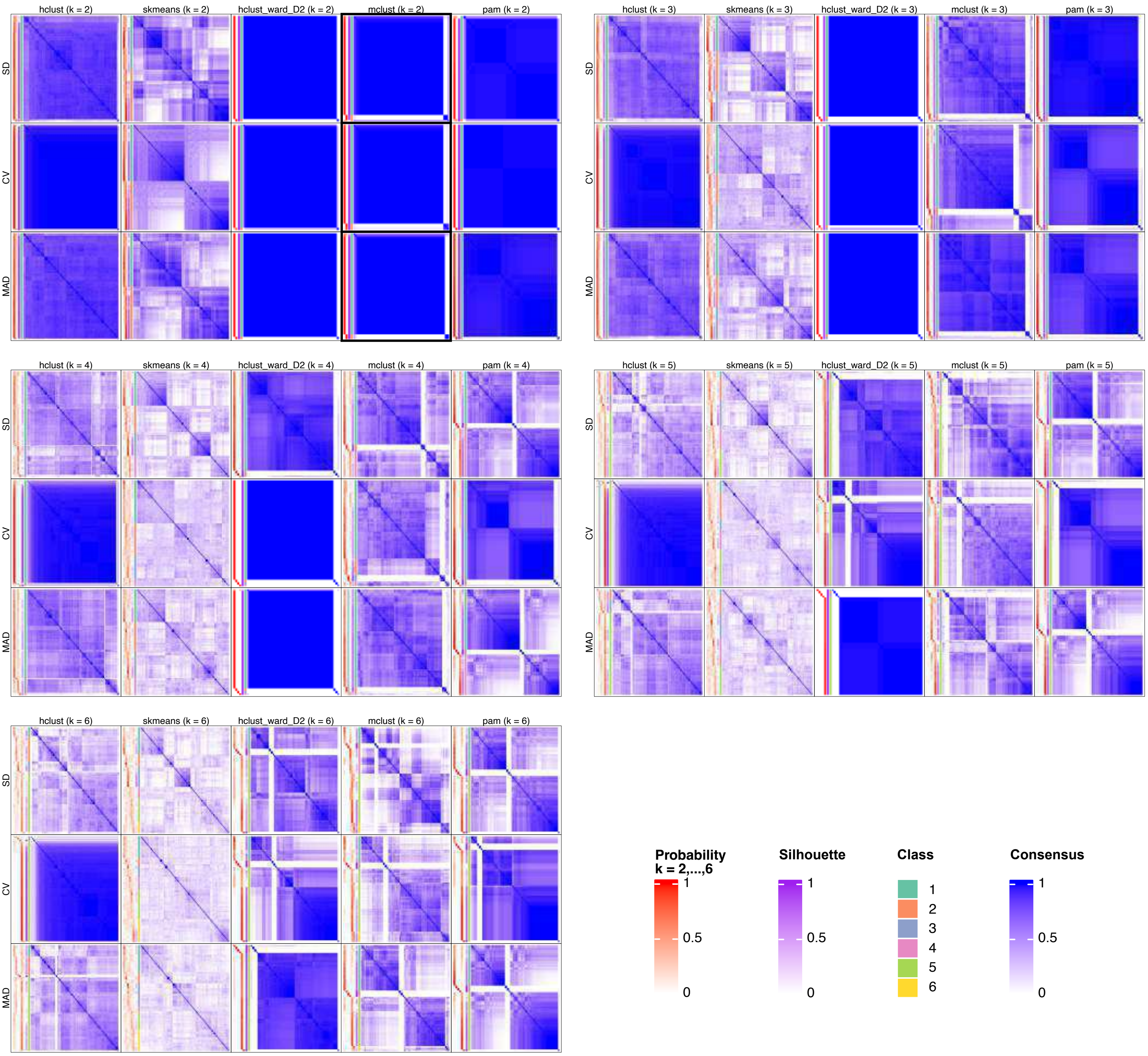

D

Primary (n = 111) and recurrent tumors (n = 22) consensus partitioning, top 10 000, 20 000, 50 000 sites

Top value method: partition method	best k	1-PAC	Mean silhouette	Concordance
SD:mclust	2	1	0.985	0.993
MAD:mclust	2	1	0.986	0.993
CV:hclust	4	0.671	0.773	0.92
CV:hclust_ward_D2	5	0.549	0.818	0.899
CV:pam	6	0.492	0.659	0.854
CV:mclust	3	0.395	0.743	0.849
MAD:hclust_ward_D2	4	0.366	0.698	0.771
SD:hclust_ward_D2	6	0.361	0.717	0.801
MAD:skmeans	2	0.338	0.718	0.868
SD:pam	6	0.323	0.618	0.749
SD:skmeans	2	0.318	0.645	0.843
MAD:pam	6	0.285	0.697	0.797
CV:skmeans	2	0.244	0.7	0.844
MAD:hclust	4	0.037	0.54	0.634
SD:hclust	4	0.03	0.611	0.73

Consensus heatmaps for k = 2,...,6

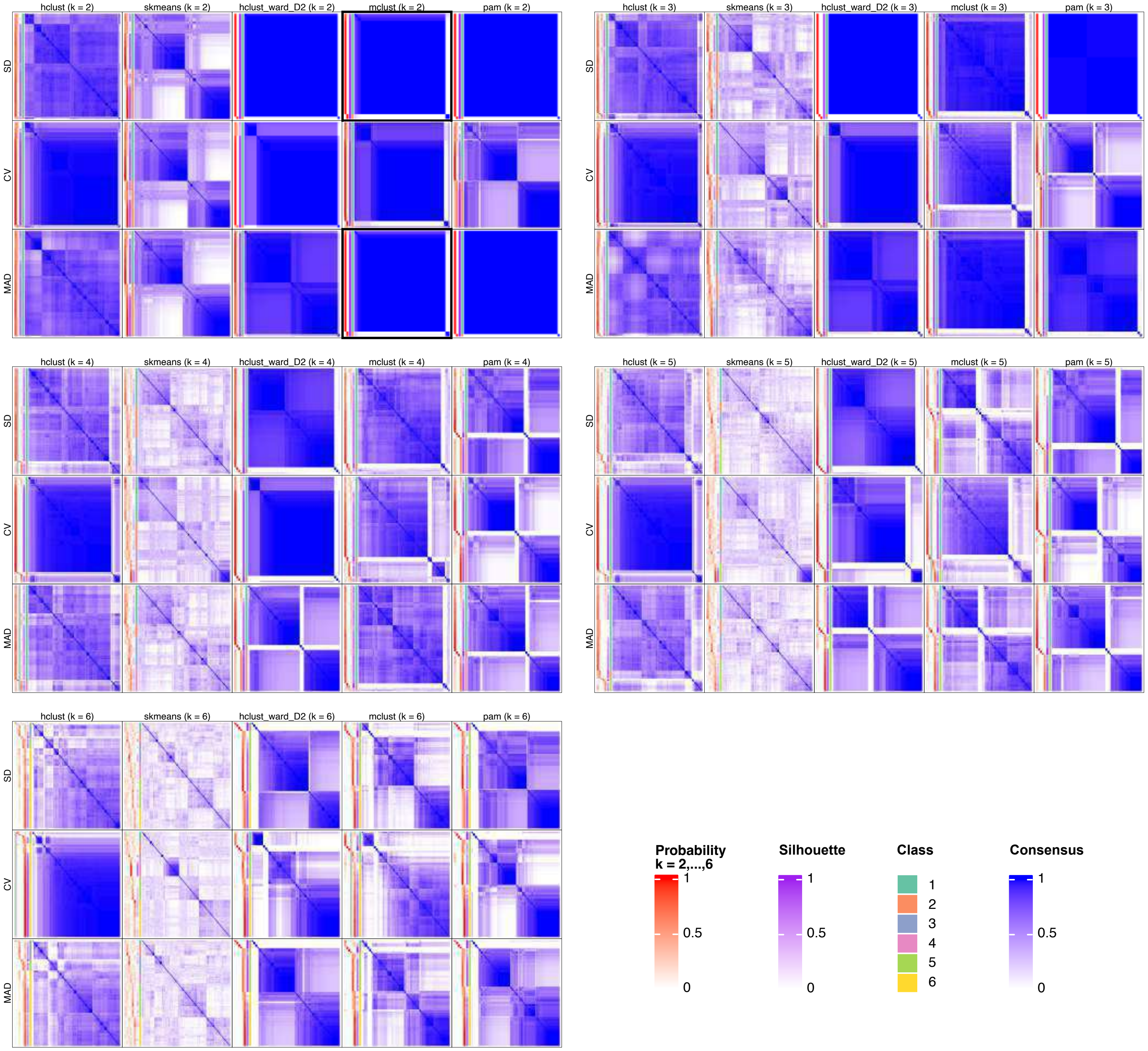

Supplementary Figure 5. No evidence of epigenetic subgroups in CN based on consensus clustering.
A) Consensus clustering of primary tumors with top 1 000, 2 000, 5 000 sites revealed a stable partitioning (1-PAC > 0.9, SD:mclust k = 2) with 2 subgroups. However, the two resulting groups consisted of four cases (#19, #65, #96, #98) on the one hand and the rest of the cohort on the other hand. The four cases did not show distinct clinical or molecular characteristics. **B)** When using the top 10 000, 20 000 and 50 000 sites for consensus clustering, we did not find a stable partitioning. CV:mclust k = 3 yielded the highest 1-PAC (0.789) and showed a good concordance (0.925). The resulting partition distinguished three classes with 90, 19, and 3 cases (Column CV:mclust k = 3). Notably, class 2 with 19 cases contained only 1/14 cases (no follow-up n = 5) which had a recurrence, compared to 20/68 (no follow-up n =22) recurring cases in class 1. **C-D)** When investigating primary and recurrent tumors (n = 133), selecting the top 1 000, 2 000, 5 000 sites (C) revealed three highly stable partitions: SD:mclust k = 2, CV:mclust k = 2 and MAD: mclustk = 2 with 8 (#6p + r, #30p + r1 + r2, #34p + r1 + r2), 8 (#19, #30p + r1 + r2, #34p + r1 + r2, #96) and 6 (#30p + r1 + r2, #34p + r1 + r2) cases in the smaller subgroups. Applying clustering to a larger set of sites (10 000, 20 0000 and 50 000, D) revealed similar results with two stable partitions for SD:mclust k = 2 and MAD:mclust k = 2 with 6 cases each (#30p + r1 + r2, #34p + r1 + r2).

A

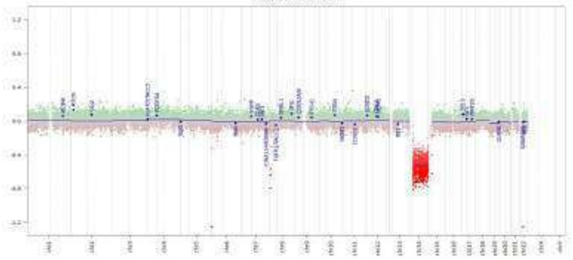

CN11 (primary): whole Chr14 loss

B

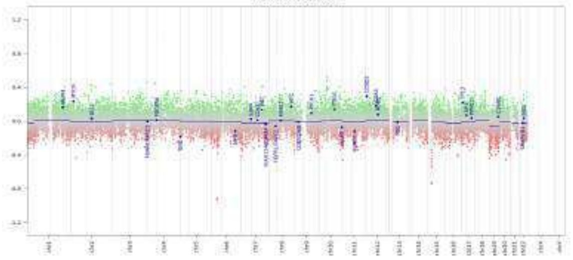

CN14 (primary): partial Chr1 p-arm loss, whole Chr19 loss

C

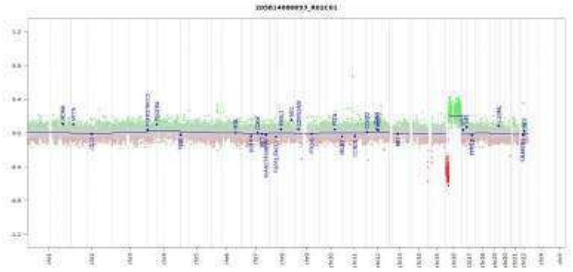

CN35 (recurrence): Chr16 p-arm loss, Chr16 q-arm gain

D

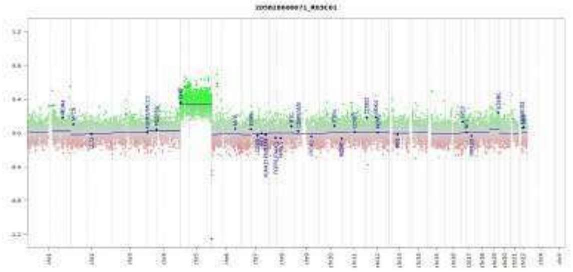

CN43 (recurrence): whole Chr5 gain

E

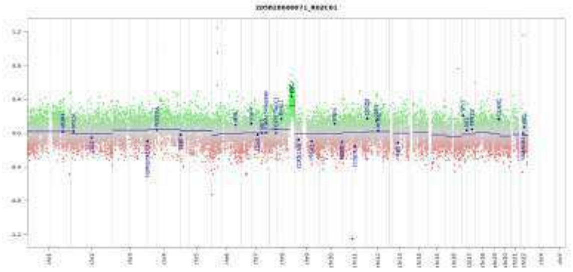

CN44 (recurrence): partial Chr8 q-arm gain

F

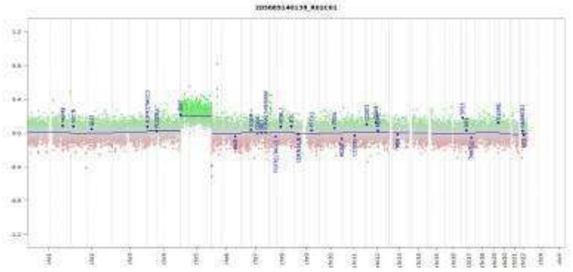

CN53 (primary): whole Chr5 gain

G

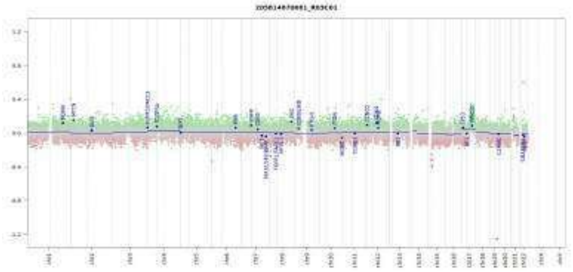

CN54 (recurrence): partial Chr17 gain

H

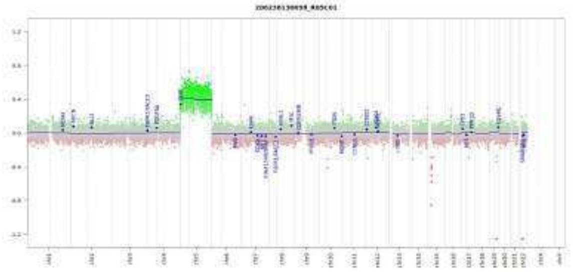

CN72 (primary): whole Chr5 gain

I

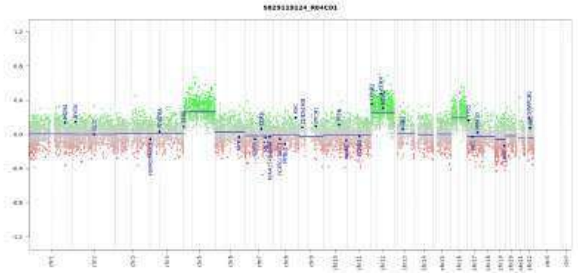

CN98 (primary): whole Chr5, Chr12, Chr16 gain

J

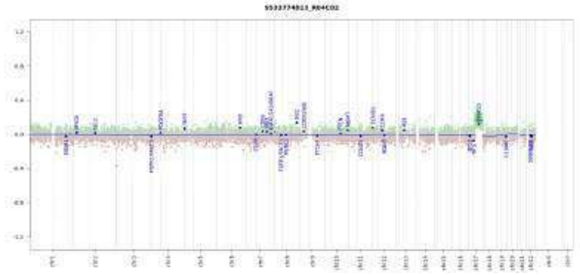

CN108 (primary): partial Chr17 q-arm gain

K

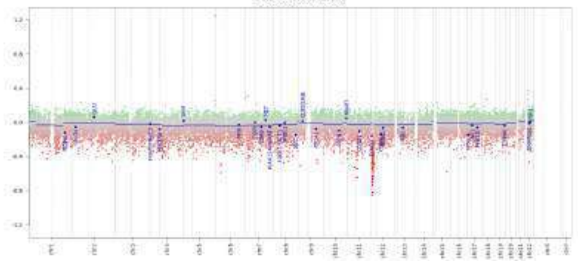

CN121 (primary): partial Chr12 p-arm loss:

M

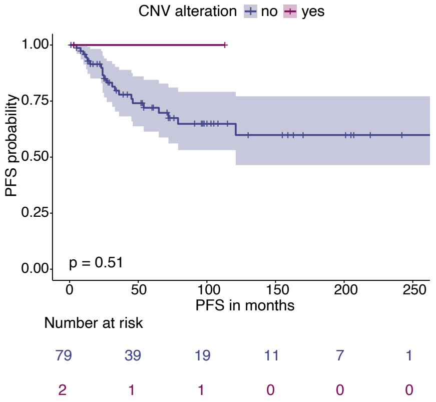

Supplementary Figure 6. CNV alterations in CNs and impact on PFS.

A-K) Chromosomal alterations were observed in 11 CN cases, four of them showed a whole chromosome 5 gain (D, F, H, I). M) For the two patients with CNV alterations and available follow-up data (CN11, A and CN72, H) no survival difference was seen compared to patients without CNV alterations in Kaplan-Meier estimation.

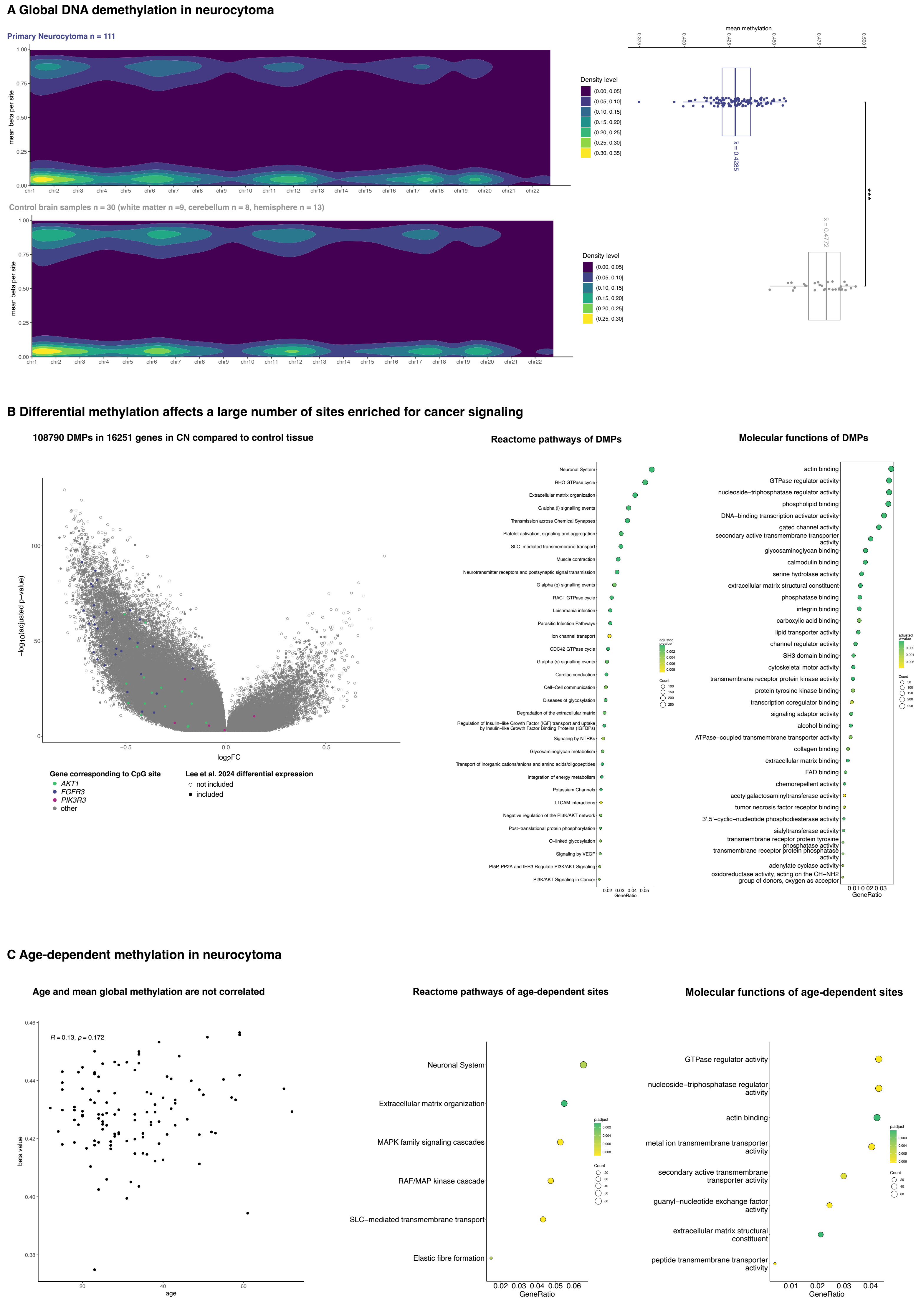

A Global DNA demethylation in neurocytoma
Primary Neurocytoma n = 111
mean beta per site
Density level
mean methylation
x̄ = 0.4285
Control brain samples n = 30 (white matter n =9, cerebellum n = 8, hemisphere n = 13)
x̄ = 0.4772
***
B Differential methylation affects a large number of sites enriched for cancer signaling
108790 DMPs in 16251 genes in CN compared to control tissue
−log10(adjusted p−value)
log2FC
Gene corresponding to CpG site
AKT1
FGFR3
PIK3R3
other
Lee et al. 2024 differential expression
not included
included
Reactome pathways of DMPs
Neuronal System
RHO GTPase cycle
Extracellular matrix organization
G alpha (i) signalling events
Transmission across Chemical Synapses
Platelet activation, signaling and aggregation
SLC−mediated transmembrane transport
Muscle contraction
Neurotransmitter receptors and postsynaptic signal transmission
G alpha (q) signalling events
RAC1 GTPase cycle
Leishmania infection
Parasitic Infection Pathways
Ion channel transport
CDC42 GTPase cycle
G alpha (s) signalling events
Cardiac conduction
Cell−Cell communication
Diseases of glycosylation
Degradation of the extracellular matrix
Regulation of Insulin−like Growth Factor (IGF) transport and uptake by Insulin−like Growth Factor Binding Proteins (IGFBPs)
Signaling by NTRKs
Glycosaminoglycan metabolism
Transport of inorganic cations/anions and amino acids/oligopeptides
Integration of energy metabolism
Potassium Channels
L1CAM interactions
Negative regulation of the PI3K/AKT network
Post−translational protein phosphorylation
O−linked glycosylation
Signaling by VEGF
PI5P, PP2A and IER3 Regulate PI3K/AKT Signaling
PI3K/AKT Signaling in Cancer
GeneRatio
Molecular functions of DMPs
actin binding
GTPase regulator activity
nucleoside−triphosphatase regulator activity
phospholipid binding
DNA−binding transcription activator activity
gated channel activity
secondary active transmembrane transporter activity
glycosaminoglycan binding
calmodulin binding
serine hydrolase activity
extracellular matrix structural constituent
phosphatase binding
integrin binding
carboxylic acid binding
lipid transporter activity
channel regulator activity
SH3 domain binding
cytoskeletal motor activity
transmembrane receptor protein kinase activity
protein tyrosine kinase binding
transcription coregulator binding
signaling adaptor activity
alcohol binding
ATPase−coupled transmembrane transporter activity
collagen binding
extracellular matrix binding
FAD binding
chemorepellent activity
acetylgalactosaminyltransferase activity
tumor necrosis factor receptor binding
3',5'−cyclic−nucleotide phosphodiesterase activity
sialyltransferase activity
transmembrane receptor protein tyrosine phosphatase activity
transmembrane receptor protein phosphatase activity
adenylate cyclase activity
oxidoreductase activity, acting on the CH−NH2 group of donors, oxygen as acceptor
GeneRatio
C Age-dependent methylation in neurocytoma
Age and mean global methylation are not correlated
R = 0.13, p = 0.172
beta value
age
Reactome pathways of age-dependent sites
Neuronal System
Extracellular matrix organization
MAPK family signaling cascades
RAF/MAP kinase cascade
SLC−mediated transmembrane transport
Elastic fibre formation
GeneRatio
p.adjust
Count
Molecular functions of age-dependent sites
GTPase regulator activity
nucleoside−triphosphatase regulator activity
actin binding
metal ion transmembrane transporter activity
secondary active transmembrane transporter activity
guanyl−nucleotide exchange factor activity
extracellular matrix structural constituent
peptide transmembrane transporter activity
GeneRatio

Supplementary Figure 7. Global DNA demethylation, extensive differential methylation, and age-dependent methylation in CN.
A) CN exhibit a significantly lower global methylation compared to pooled control samples and hypomethylation affected methylated regions across the genome. **B)** Affected genes were enriched in *RHO GTPase* cycle, extracellular matrix organization, *NTRK* signaling, as well as in *PI3K/AKT* pathways. **C)** Global methylation was not correlated with age. Age-dependent sites affected genes involved in the neuronal system, as well as *MAPK* signaling.

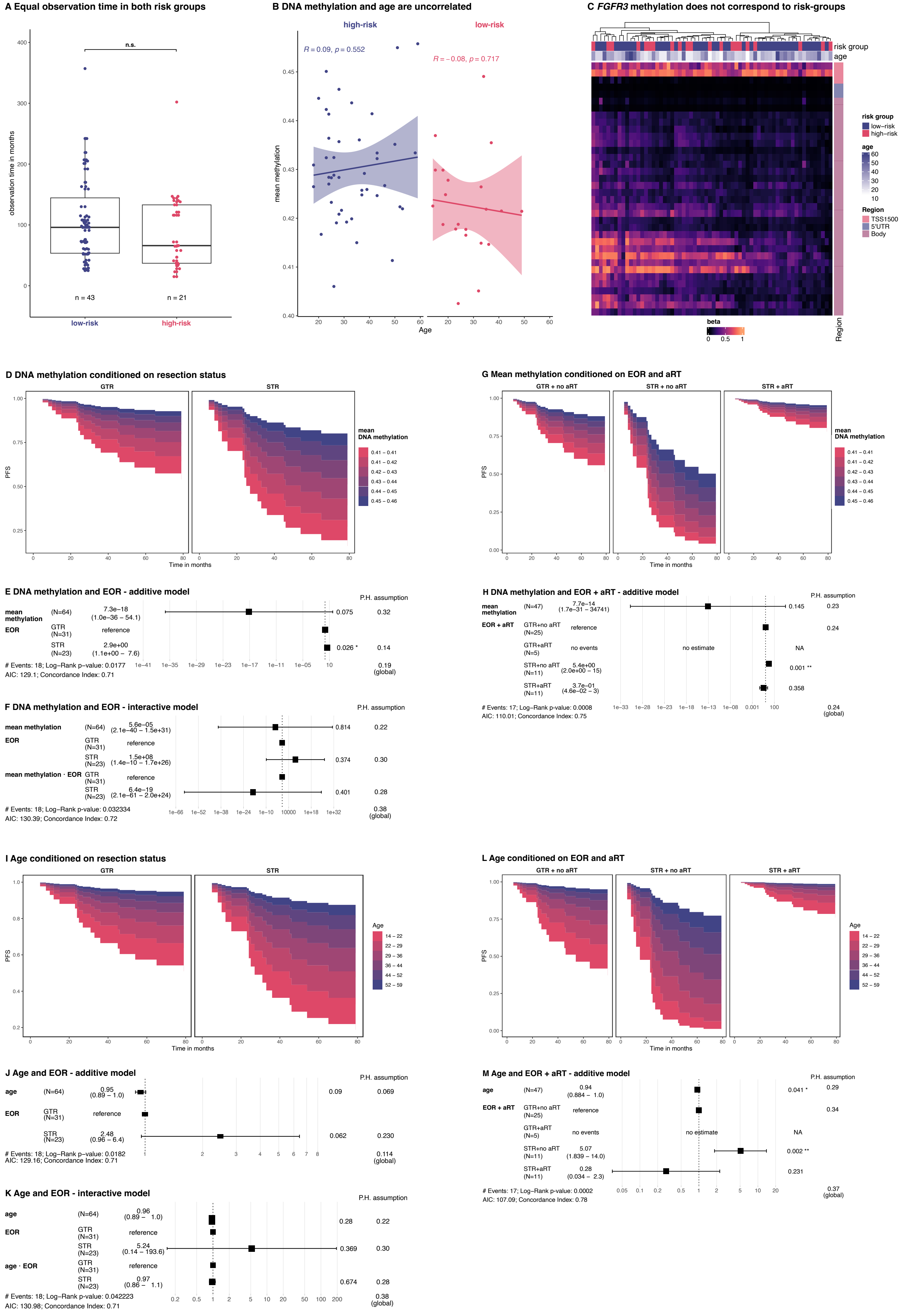

A Equal observation time in both risk groups
n.s.
observation time in months
n = 43
n = 21
low-risk
high-risk
B DNA methylation and age are uncorrelated
high-risk
low-risk
R = 0.09, p = 0.552
R = −0.08, p = 0.717
mean methylation
Age
C FGFR3 methylation does not correspond to risk-groups
risk group
age
risk group
low−risk
high−risk
age
Region
TSS1500
5'UTR
Body
beta
Region
D DNA methylation conditioned on resection status
GTR
STR
PFS
Time in months
mean
DNA methylation
0.41 – 0.41
0.41 – 0.42
0.42 – 0.43
0.43 – 0.44
0.44 – 0.45
0.45 – 0.46
G Mean methylation conditioned on EOR and aRT
GTR + no aRT
STR + no aRT
STR + aRT
E DNA methylation and EOR - additive model
P.H. assumption
mean methylation (N=64) 7.3e−18 (1.0e−36 – 54.1) 0.075 0.32
EOR GTR (N=31) reference
STR (N=23) 2.9e+00 (1.1e+00 – 7.6) 0.026 * 0.14
Events: 18; Log−Rank p-value: 0.0177
AIC: 129.1; Concordance Index: 0.71
0.19 (global)
H DNA methylation and EOR + aRT - additive model
P.H. assumption
mean methylation (N=47) 7.7e−14 (1.7e−31 – 34741) 0.145 0.23
EOR + aRT GTR+no aRT (N=25) reference 0.24
GTR+aRT (N=5) no events no estimate NA
STR+no aRT (N=11) 5.4e+00 (2.0e+00 – 15) 0.001 **
STR+aRT (N=11) 3.7e−01 (4.6e−02 – 3) 0.358
Events: 17; Log−Rank p-value: 0.0008
AIC: 110.01; Concordance Index: 0.75
0.24 (global)
F DNA methylation and EOR - interactive model
P.H. assumption
mean methylation (N=64) 5.6e−05 (2.1e−40 – 1.5e+31) 0.814 0.22
EOR GTR (N=31) reference
STR (N=23) 1.5e+08 (1.4e−10 – 1.7e+26) 0.374 0.30
mean methylation · EOR GTR (N=31) reference
STR (N=23) 6.4e−19 (2.1e−61 – 2.0e+24) 0.401 0.28
Events: 18; Log−Rank p-value: 0.032334
AIC: 130.39; Concordance Index: 0.72
0.38 (global)
I Age conditioned on resection status
GTR
STR
PFS
Time in months
Age
14 – 22
22 – 29
29 – 36
36 – 44
44 – 52
52 – 59
L Age conditioned on EOR and aRT
GTR + no aRT
STR + no aRT
STR + aRT
J Age and EOR - additive model
P.H. assumption
age (N=64) 0.95 (0.89 – 1.0) 0.09 0.069
EOR GTR (N=31) reference
STR (N=23) 2.48 (0.96 – 6.4) 0.062 0.230
Events: 18; Log−Rank p-value: 0.0182
AIC: 129.16; Concordance Index: 0.71
0.114 (global)
M Age and EOR + aRT - additive model
P.H. assumption
age (N=47) 0.94 (0.884 – 1.0) 0.041 * 0.29
EOR + aRT GTR+no aRT (N=25) reference 0.34
GTR+aRT (N=5) no events no estimate NA
STR+no aRT (N=11) 5.07 (1.839 – 14.0) 0.002 **
STR+aRT (N=11) 0.28 (0.034 – 2.3) 0.231
Events: 17; Log−Rank p-value: 0.0002
AIC: 107.09; Concordance Index: 0.78
0.37 (global)
K Age and EOR - interactive model
P.H. assumption
age (N=64) 0.96 (0.89 – 1.0) 0.28 0.22
EOR GTR (N=31) reference
STR (N=23) 5.24 (0.14 – 193.6) 0.369 0.30
age · EOR GTR (N=31) reference
STR (N=23) 0.97 (0.86 – 1.1) 0.674 0.28
Events: 18; Log−Rank p-value: 0.042223
AIC: 130.98; Concordance Index: 0.71
0.38 (global)

Supplementary Figure 8. Risk group characteristics and stratification potential of age and DNA methylation. **A)** Observation times between high- and low-risk groups were equal. **B)** DNA methylation and age were not correlated within the risk groups. **C)** FGFR3 DNA methylation levels did not cluster according to risk groups or age. **D-H)** DNA methylation does not provide a significant stratification criterion when accounting for resection status (P.H. – proportional hazard) or resection status and radiotherapy. **I-K)** When accounting for resection status, age is borderline non-significant in predicting PFS in an additive model, and non-significant in an interactive model. **L-M)** On the contrary, age was predictive in an additive model with the combined treatment regimen. Due to the risk of overfitting, we did not test for an interactive effect of age, DNA methylation, and treatment regimen.

A

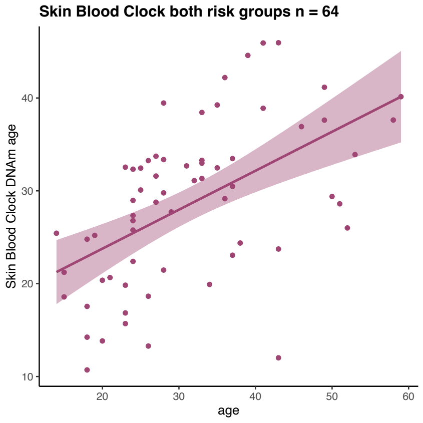

	Skin Blood Clock, 361 CpG sites	
DNAm age	0.69 ***	(0.14)
MAE	7.26	
adjusted R²	0.28	
Log Likelihood	-233.36	
AIC	472.72	
AIC (#CpG as parameter)	1188.72	

B

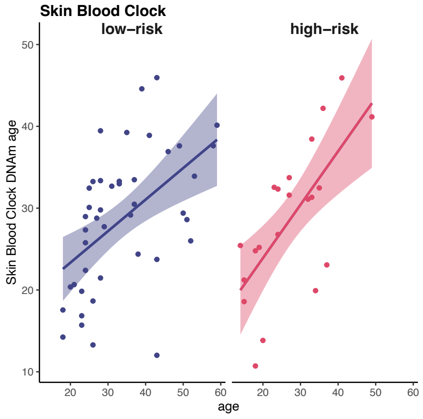

	Skin Blood Clock, 361 CpG sites	
DNAm age	0.69 ***	(0.13)
risk-group	-6.34 *	(2.40)
**MAE**	**7.11**	
**adjusted R²**	**0.34**	
**Log Likelihood**	**-229.88**	
**AIC**	**467.76**	
AIC (#CpG as parameter)	1181.76	

C

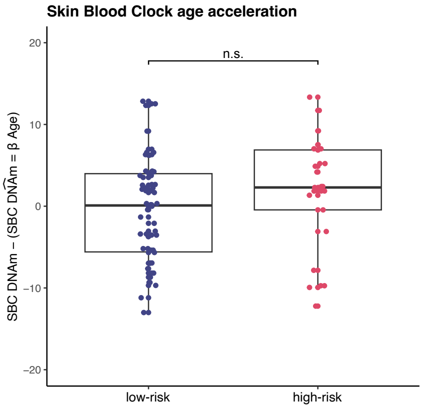

	Skin Blood Clock DNAm predicted by age	
age	0.42 ***	(0.08)
MAE	5.88	
adjusted R²	0.28	
Log Likelihood	-217.34	
AIC	440.68	

D

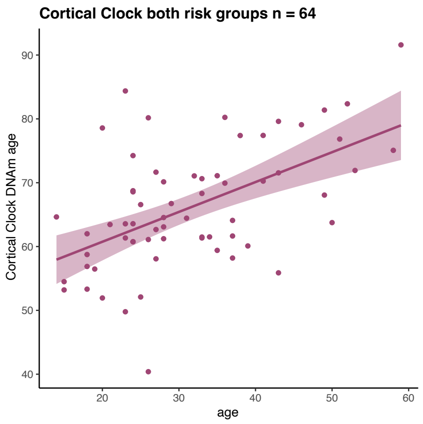

	Cortical Clock, 311 CpG sites	
DNAm age	0.63 ***	(0.12)
MAE	7.46	
adjusted R²	0.28	
Log Likelihood	-233.18	
AIC	472.35	
AIC (#CpG as parameter)	1088.35	

E

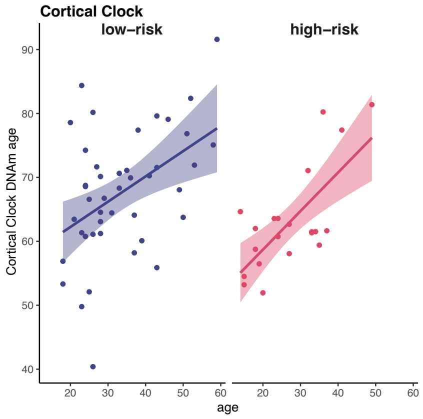

	Cortical Clock, 311 CpG sites	
DNAm age	0.59 ***	(0.13)
risk-group	-3.61	(2.55)
MAE	7.35	
adjusted R²	0.29	
Log Likelihood	-232.14	
AIC	472.27	
**AIC (#CpG as parameter)**	**1086.27**	

F

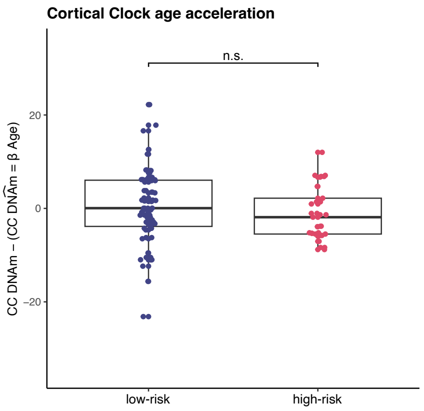

	Cortical Clock DNAm predicted by age	
age	0.47 ***	(0.09)
MAE	6.15	
adjusted R²	0.28	
Log Likelihood	-223.62	
AIC	453.23	

G

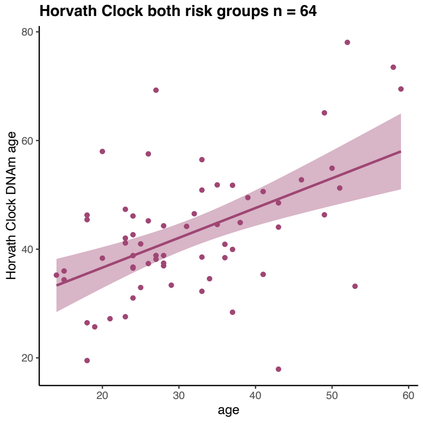

	Horvath Clock, 319 CpG sites	
DNAm age	0.47 ***	(0.10)
MAE	7.61	
adjusted R²	0.25	
Log Likelihood	-234.81	
AIC	475.61	
AIC (#CpG as parameter)	1107.61	

H

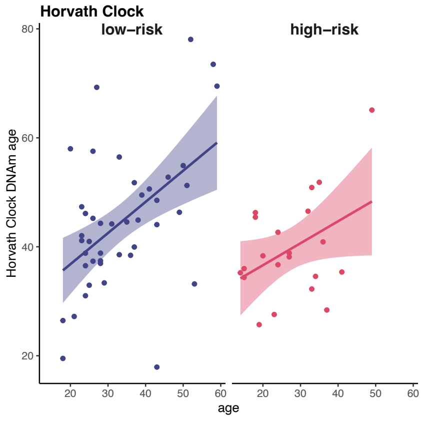

	Horvath Clock, 319 CpG sites	
DNAm age	0.44 ***	(0.10)
risk-group	-4.07	(2.59)
MAE	7.55	
adjusted R²	0.26	
Log Likelihood	-233.53	
AIC	475.06	
AIC (#CpG as parameter)	1105.06	

I

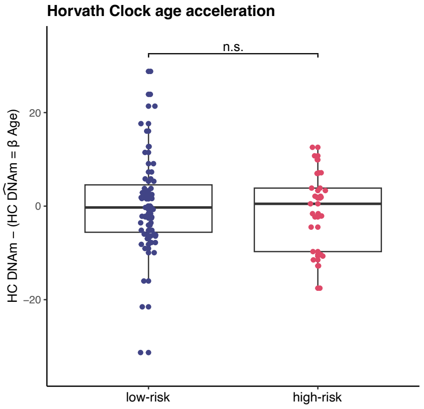

	Horvath Clock DNAm predicted by age	
age	0.55 ***	(0.12)
MAE	7.57	
adjusted R²	0.25	
Log Likelihood	-239.72	
AIC	485.45	

Supplementary Figure 9. DNAm age and acceleration.
Predicted DNAm age and acceleration (residuals of DNAmage predicted by age) in Skin and Blood Clock **(A-C),** Cortical Clock **(D-F),** and Horvath Clock **(G-I).** Linear models to predict the chronological age using DNAm age are depicted below with the standard error of the coefficient estimates in brackets. For each clock, we report the number of CpG sites in our dataset. Best metrics for DNAm age prediction in bold. Horvath Clock and Cortical Clock showed no significant improvement upon including risk group (Likelihood-Ratio-Test: X^2 = 2.5499, p-value = 0.1103 and X^2 = 2.0801, p-value = 0.1492), however, the Skin and Blood Clock showed significance (X^2 = 6.9605, p-value = 0.0083). *** - p < 0.001; * - p < 0.05, MAE - mean absolute error, AIC -Akaike Information Criterion.

A Integrated model: calibration at 3 Year Prediction

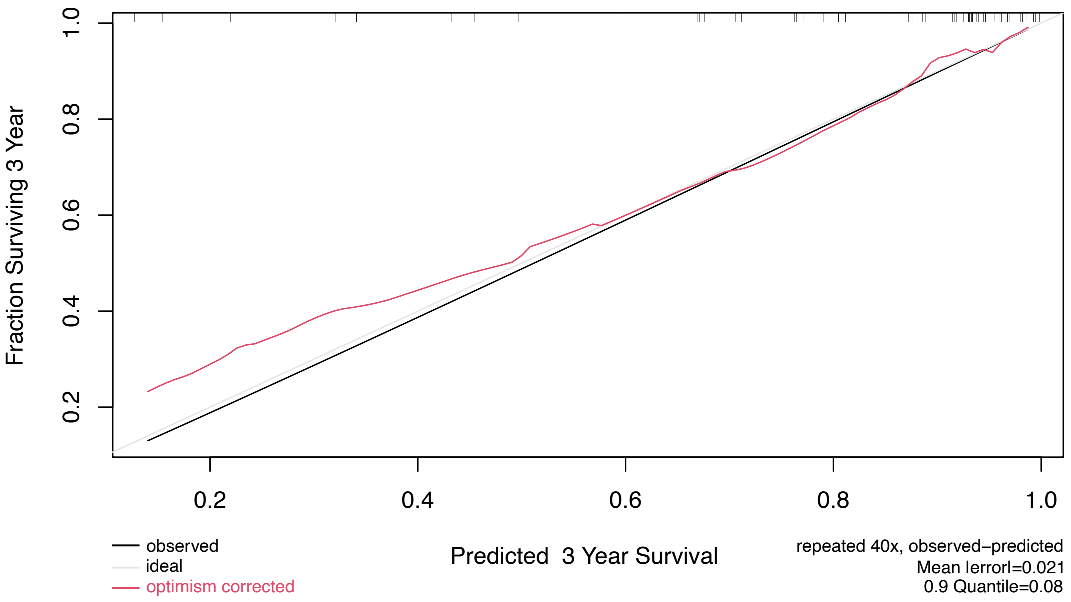

B Integrated model: calibration at 5 Year Prediction

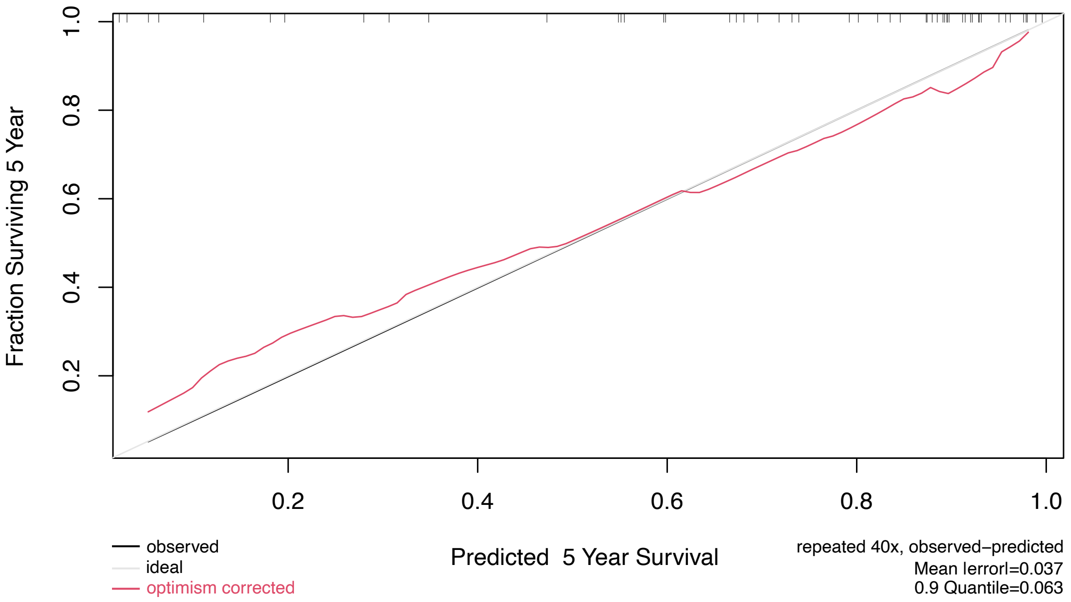

C Integrated model: internal validation

10-fold cross-validation, repeated 100 times	
C-index	0.78
Somer's D_{xy}	-0.57
Nagelkerke R^2	0.31
Slope	0.79
Discrimination index	0.12
Unreliability index	0.04
Quality (D - U)	0.07
Gini's mean difference	0.72

Supplementary Figure 10. Calibration and Validation of the integrated model.
A) Calibration of the integrated model's (Figure 4G) predictions at 3 years based on 40 repetitions**. B)** Calibration of the model's predictions (Figure 4G) at 5 years based on 40 repetitions. **C)** Results of internal 10-fold cross-validation using 100 repetitions.
