# Supplementary material for: Outcome-associated factors in a molecularly defined cohort of central neurocytoma
Source: Acta Neuropathol. 2025 Jun 11;149(1):61. doi: 10.1007/s00401-025-02894-3 (PMC12158839; doi:10.1007/s00401-025-02894-3)
Supplement: Supplementary file 3 — (PDF 276 KB) [file 401_2025_2894_MOESM3_ESM.pdf]

Supplementary Table 2. Univariate survival analysis of histopathological and clinical variables.

Variable	Cases	Number of events	Kaplan Meier model	Cox proprotional hazard model				
			log rank t-test p-value	p-value	CI	C-index	HR	test for PH p-value
Global Ki67-index cutoff 2%*	61	17	0.0055	0.0095	1.3920 – 10.7821	0.6473	3.8741	0.0586
Focal Ki67-index cutoff 2%*	61	17	0.0050	0.0112	1.4478 – 17.8469	0.6953	5.0833	0.059
Focal Ki67-index cutoff 3%*	61	17	0.0171	0.023	1.1690 – 8.2420	0.6217	3.1040	0.0914
Global Ki67-index (continuous) *	61	17	-	0.0042	1.1333 – 1.9487	0.7573	1.4861	0.0482
Focal Ki67-index (continuous) *	61	17	-	0.0243	1.0535 – 1.4204	0.7101	1.2233	0.0586
≥ 1.5 mitosis per mm^2 *	70	18	0.0460	0.0539	0.9846 – 6.5870	0.6183	2.5467	0.1533
≥ 1.9 mitosis per mm^2 *	70	18	1.6E-05	0.0002	2.4949 – 18.2034	0.6669	6.7391	0.0062
≥ 2.3 mitosis per mm^2 *	70	18	0.0004	0.0015	1.9332 – 16.0818	0.6229	5.5757	0.0273
≥ 2.7 mitosis per mm^2 *	70	18	0.0154	0.0247	1.2007 – 14.7116	0.5703	4.2029	0.0914
Mitosis per mm^2 (continuous)*	70	18	-	0.0243	1.0580 – 2.2573	0.6550	1.5454	0.0914
Necrosis*	70	18	0.2600	0.9981	0 - NA	0.5329	0.0000	1.0000
Vascular proliferation*	70	18	0.4200	0.4336	0.0589 – 3.3710	0.5191	0.4455	0.3500
Diagnosis: CN vs. aCN (≥one atypical feature)*	70	18	0.4625	0.4639	0.5517 – 3.6865	0.5631	1.4261	0.6866
Diagnosis: CN vs. aCN (≥two atypical features)*	70	18	0.7638	0.7643	0.0949 – 5.6399	0.4967	0.7316	0.9122
Calcification	71	18	0.0980	0.1188	0.0713 – 1.3500	0.5840	0.3102	0.4900
Location (one vs. multiple ventricles)	79	29	0.5200	0.5223	0.3035 – 1.8321	0.5069	0.7457	0.1700
Contrast enhancement	48	18	0.5900	0.5926	0.3421 – 6.5415	0.5393	1.4960	0.4100

* Central review
CI: Confidence Intervall
C-index: Concordance index
HR: Hazard ratio
PH: Proportional hazard

Supplementary Table 2. Univariate survival analysis of histopathological and clinical variables.

Variable	Cases	Number of events	Kaplan Meier model	Cox proprotional hazard model				
			log rank t-test p-value	p-value	CI	C-index	HR	test for PH p-value
Extent of resection (GTR vs. STR)	76	26	**0.0470**	0.0514	0.9952 – 4.6750	0.6093	2.1570	0.3100
Adjuvant radiation (aRT)	75	25	**0.0190**	**0.0298**	0.0784 – 0.8769	0.6145	0.2622	0.5700
aRT after GTR	44	12	0.3700	0.3833	0.0517 – 3.1210	0.5608	0.4018	0.2500
aRT after STR	30	12	**0.0009**	**0.0052**	0.0242-0.5206	0.7351	0.1122	0.7300
CNV alteration	81	21	0.51	0.9972	0 – NA	0.5097	1.08E-07	1.08E-09
CNV load	75	21	-	0.2492	1 – 1	0.5797	1.0000	0.1000
FGFR3 expression	48	14	0.0910	0.1031	0.1168 – 1.2183	0.5841	0.3773	0.1600

* Central review
CI: Confidence Intervall
C-index: Concordance index
HR: Hazard ratio
PH: Proportional hazard
