# Supplementary material for: Outcome-associated factors in a molecularly defined cohort of central neurocytoma
Source: Acta Neuropathol. 2025 Jun 11;149(1):61. doi: 10.1007/s00401-025-02894-3 (PMC12158839; doi:10.1007/s00401-025-02894-3)
Supplement: Supplementary file 7 — (PDF 144 KB) [file 401_2025_2894_MOESM7_ESM.pdf]

Supplementary Table 6. Patient characteristics risk groups

	Low-risk group	%	High-risk group (n)	%
**Total**	43		21	
**Age**				
Median	29y		27y	
Range	18 – 59y		14 – 49y	
		W = 396, **p = 0.0018**		
**Sex**				
Female	20	47%	13	62%
Male	23	53%	8	38%
	p = 0.7608		p = 0.3833	
		p = 0.2941		
Location				
One ventricle	22	56%	15	75%
Biventricular	6	15%	1	5%
III. ventricle	1	3%	0	0%
Lateral + III. ventricle	6	15%	3	15%
IV. ventricle	0	0%	0	0%
Intraventricular	4	10%	1	5%
NA	4		1	
		p = 0.7398		
Radiological characteristics				
**Contrast enhancement**				
Yes	18	82%	13	87%
No	4	18%	2	13%
NA	21		7	
		p = 1		
**Hydrocephalus**				
Yes	11	38%	3	21%
No	18	62%	11	79%
NA	14		7	
		p = 0.3239		
**Leptomeningeal dissimination**				
Yes	0		1	7%
No	24	100%	13	93%
NA	19		7	
		p = 0.3684		
Therapy				
**Extent of resection**				
GTR	24	67%	7	39%
STR	12	33%	11	61%
NA	7		3	
		p = 0.0798		
**Adjuvant radiotherapy**				
Yes	15	42%	1	6%
No	21	58%	16	94%
NA	7		4	
		**p = 0.0098**		
FGFR3				
moderate	11	41%	10	71%
strong	15	56%	4	29%
negative	1	4%	0	0%
NA	16		7	
		p = 0.1325		
