# Supplementary material for: Outcome-associated factors in a molecularly defined cohort of central neurocytoma
Source: Acta Neuropathol. 2025 Jun 11;149(1):61. doi: 10.1007/s00401-025-02894-3 (PMC12158839; doi:10.1007/s00401-025-02894-3)
Supplement: Supplementary file 10 — (DOCX 57 KB) [file 401_2025_2894_MOESM10_ESM.docx]

**Supplementary Methods 1. Immunohistochemical procedures and histopathological review**

Immunohistochemistry was performed using the standard antigen retrieval techniques (CC1 buffer, pH8.0, Ventana Medical Systems) on the BenchMark ULTRA (SN319330, SN319325, Ventana Medical Systems) or the Leica Bond-III (SN3212926, S3211649, Leica Biosystems) platforms. Primary antibodies used in this study included monoclonal mouse anti-MIB-1 (Ki67, 1:100, clone M7240, Dako) and monoclonal mouse anti-FGFR3 (1:200, clone B9, Santa Cruz Biotechnologies). Stained slides were digitised using the Aperio GT 450 Dx slide Scanner (SN5545086) and subsequently reviewed by a board-certified neuropathologist.

For evaluating inter-rater variability of the current histopathological stratification criteria Ki67 index and atypical features (mitotic count, necrosis, vascular proliferation), 30 CN cases were reviewed by eight respectively five board-certified neuropathologists. To compare the Ki67 index estimates, raters were instructed, to first estimate the overall (total) Ki67 index of the whole slide and then to identify the area with the highest focal KI67 index (“hotspot” region) and the corresponding value. For the calculation of the ICC3, Ki67 values were simplified as follows: If the Ki67 index was described with “</>” a value one below or one above was chosen, in case of “<1%” the value was set to “0.5%”. If the Ki67 index was reported as a value between two percentages e.g. “3% - 4%”, the value was set to the average of the two percentages given (e.g. 3.5%).

Inter-rater agreement among individual raters was assessed by calculating Krippendorff's alpha. For the continuous variables Ki67 and mitotic count, overall agreement was analyzed using the intraclass correlation coefficient based on single ratings, absolute agreement, and a two-way mixed effects model (ICC3). For the nominal variables necrosis and vascular proliferation, Krippendorff's alpha was employed. Both analyses were performed with R version 4.4.0, using the packages irr v0.84.1 and corrplot v0.94 for visualization. ICC3 scores were interpreted as follows ^1^: values below 0.5 indicate poor interrater reliability, values from 0.5 to 0.75 indicate moderate reliability, values from 0.75 to 0.9 indicate good reliability, and values above 0.9 indicate excellent reliability. For Krippendorff's alpha, values greater than 0.8 were considered reliable, values between 0.67 and 0.79 were considered moderately reliable, and values less than 0.67 were considered unreliable. Alpha values less than 0 indicate systematic disagreement ^2^.

To investigate the variability of Ki67 staining methods, immunohistochemical stains of seven CN cases were performed in the laboratories of six different neuropathological institutions (Berlin, Frankfurt, Hamburg, Hannover, Leipzig, Münster), and information on staining methods (antibody, dilution, staining machine) were collected. After digitization, slides were evaluated within the software QuPath (v0.5.1). To assess the variation between the differently stained slides within a case, an area of 0.1mm^2^ sized was selected and the Ki67 index was determined by manual counting. Cells with a light brown to dark brown appearance were counted as positive, dark blue to light grayish cells were counted as negative. Cells that overlapped with the annotation box were included in the count if at least 50% of the cell was within the box. Results were then compared between the slides within a case.

**Supplementary Methods 2. DNA Methylation and CNV analysis**

For the analysis of DNA methylation data probes with p-values ≥ 0.01 in 90% of the samples, probes located on sex chromosomes, and cross-reactive probes ^3^ were removed before beta-value calculation. In addition, probes with poor correlation between both array types and absolute differences ≥ 0.1 in beta-values ^4^ were discarded. Filtering resulted in 339,381 sites for the n = 111 primary tumors and 339,394 sites for the n = 133 primary and recurrent tumors. For the t-SNE analysis, we followed the methodology of Capper et al. (2018) ^5^. We applied MNP preprocessing (available at https://github.com/mwsill/mnp_training/blob/master/preprocessing.R) to the reference cohort and selected the 37,000 sites with the highest standard deviation. We then intersected these sites with those remaining after filtering the CN sites. The first 94 singular value decomposition (SVD) components derived from these sites were used for t-SNE analysis using the R package Rtsne v0.17, with parameters set to theta = 0, perplexity = 54 (calculated as the nearest integer to the square root of the number of samples), and 2000 iterations.

All combinations (n = 15) of the following feature selection and clustering methods were employed to investigate the stability of k = 2-6 subgroups. Sites were selected based on standard deviation (SD), coefficient of variance (CV), and median absolute deviation (MAD), selecting top 1000, 2000 and 5000 features as well as 10,000, 20,000 and 50,000 features. Clustering was conducted using hierarchical clustering with complete linkage (hclust) and Ward’s D2 method (hclust_ward_D2), spherical k-means clustering (skmeans), partitioning among medoids (pam) and model-based clustering (mclust). To test whether the resulting partitions are stable, we applied the proportion of ambiguous clustering (1-PAC) as proposed by Gu ^6^.

To test for differentially methylated positions, we applied linear models (lmFit, limma v3.58.1) followed by empirical Bayes statistics for differential expression (ebayes). We used the UCSC annotation within the 450K/EPIC arrays to retrieve gene symbols and mapped these symbols to EntrezIDs. Subsequently, we employed clusterProfiler ^7^ to find enriched Reactome Pathways ^8^ and molecular functions using an Benjamini-Hochberg adjusted p-value of 0.01 as a cut-off. For the Reactome Pathway analysis of differentially methylated positions between CN and control tissue, we additionally filtered for terms with a minimum gene set size of 100.

To compare DNA methylation between risk groups, we used a support vector machine with a linear kernel and a constant cost (C = 1) as implemented in caret v7.0-1. 70% of the data was used for training and 30% for validation.

DNA methylation age was calculated using the classic Horvath and Horvath Skin and Blood clock via methylclock v1.12.0 without cell type correction. The Cortical Clock was implemented using the documentation available via github.com/gemmashireby/CorticalClock/ ^19^. Control cortex tissue with age information (median 27 years, range 15 – 65 years) was retrieved from Kozlenkov et al. ^20^ and processed using the same pipeline as CN methylation data. DNA methylation age acceleration was estimated using residuals from age prediction models.

**Supplementary Methods 3. Statistical tests**

For continuous data, we applied t-tests if the data followed a homoscedastic Gaussian distribution and Mann-Whitney-U-test for other distributions. For categorical data with two variables, we used a two-sided Fisher’s Exact test and a two-sided binomial test for one categorical variable.

Literature:

**1.** Koo TK, Li MY. A Guideline of Selecting and Reporting Intraclass Correlation Coefficients for Reliability Research. *J Chiropr Med.* 2016; 15(2):155-163.

**2.** Krippendorff K. Content Analysis: An Introduction to Its Methodology 2019; 4th Edition:356.

**3.** Chen YA, Lemire M, Choufani S, et al. Discovery of cross-reactive probes and polymorphic CpGs in the Illumina Infinium HumanMethylation450 microarray. *Epigenetics.* 2013; 8(2):203-209.

**4.** Fernandez-Jimenez N, Allard C, Bouchard L, et al. Comparison of Illumina 450K and EPIC arrays in placental DNA methylation. *Epigenetics.* 2019; 14(12):1177-1182.

**5.** Capper D, Jones DTW, Sill M, et al. DNA methylation-based classification of central nervous system tumours. *Nature.* 2018; 555(7697):469-474.

**6.** Gu Z, Schlesner M, Hübschmann D. cola: an R/Bioconductor package for consensus partitioning through a general framework. *Nucleic Acids Res.* 2021; 49(3):e15.

**7.** Wu T, Hu E, Xu S, et al. clusterProfiler 4.0: A universal enrichment tool for interpreting omics data. *Innovation (Camb).* 2021; 2(3):100141.

**8.** Yu G, He QY. ReactomePA: an R/Bioconductor package for reactome pathway analysis and visualization. *Mol Biosyst.* 2016; 12(2):477-479.
